# Gene‐set and multivariate genome‐wide association analysis of oppositional defiant behavior subtypes in attention‐deficit/hyperactivity disorder

**DOI:** 10.1002/ajmg.b.32346

**Published:** 2015-07-16

**Authors:** Marcel Aebi, Marjolein M. J. van Donkelaar, Geert Poelmans, Jan K. Buitelaar, Edmund J. S. Sonuga‐Barke, Argyris Stringaris, IMAGE consortium, Stephen V. Faraone, Barbara Franke, Hans‐Christoph Steinhausen, Kimm J. E. van Hulzen

**Affiliations:** ^1^ Department of Forensic Psychiatry, Child and Youth Forensic ServiceUniversity Hospital of PsychiatryZurichSwitzerland; ^2^ Department of Child and Adolescent PsychiatryUniversity of ZurichZurichSwitzerland; ^3^ Department of Human GeneticsRadboud University Medical Center, Donders Institute for Brain, Cognition and BehaviourNijmegenThe Netherlands; ^4^ Department of Cognitive NeuroscienceDonders Institute for Brain, Cognition and Behaviour, Radboud University Medical CenterNijmegenThe Netherlands; ^5^ Department of Molecular Animal PhysiologyDonders Institute for Brain, Cognition and Behavior, Radboud Institute for Molecular Life Sciences, Radboud UniversityNijmegenThe Netherlands; ^6^ Developmental Brain‐Behaviour LaboratoryDepartment of PsychologyUniversity of SouthamptonSouthamptonUK; ^7^ Department of Experimental Clinical and Health PsychologyGhent UniversityGhentBelgium; ^8^ Institute of PsychiatryKing's College LondonLondonUK; ^9^ Department of PsychiatrySUNY Upstate Medical UniversitySyracuseNew York; ^10^ Departmentof Neuroscience and PhysiologySUNY Upstate Medical UniversitySyracuseNew York; ^11^ Department of BiomedicineK.G. Jebsen Centre for Psychiatric DisordersUniversity of BergenBergenNorway; ^12^ Department of PsychiatryDonders Institute for Brain, Cognition and Behaviour, Radboud University Medical CenterNijmegenThe Netherlands; ^13^ Department of Psychology, Clinical Psychology and EpidemiologyUniversity of BaselBaselSwitzerland; ^14^ Research Unit for Child and Adolescent Psychiatry, Psychiatric HospitalAalborg University HospitalAalborgDenmark

**Keywords:** ODD, GWAS, irritability, β‐catenin, neurite outgrowth

## Abstract

Oppositional defiant disorder (ODD) is a frequent psychiatric disorder seen in children and adolescents with attention‐deficit‐hyperactivity disorder (ADHD). ODD is also a common antecedent to both affective disorders and aggressive behaviors. Although the heritability of ODD has been estimated to be around 0.60, there has been little research into the molecular genetics of ODD. The present study examined the association of irritable and defiant/vindictive dimensions and categorical subtypes of ODD (based on latent class analyses) with previously described specific polymorphisms (*DRD4* exon3 VNTR, 5‐HTTLPR, and seven *OXTR* SNPs) as well as with dopamine, serotonin, and oxytocin genes and pathways in a clinical sample of children and adolescents with ADHD. In addition, we performed a multivariate genome‐wide association study (GWAS) of the aforementioned ODD dimensions and subtypes. Apart from adjusting the analyses for age and sex, we controlled for “parental ability to cope with disruptive behavior.” None of the hypothesis‐driven analyses revealed a significant association with ODD dimensions and subtypes. Inadequate parenting behavior was significantly associated with all ODD dimensions and subtypes, most strongly with defiant/vindictive behaviors. In addition, the GWAS did not result in genome‐wide significant findings but bioinformatics and literature analyses revealed that the proteins encoded by 28 of the 53 top‐ranked genes functionally interact in a molecular landscape centered around Beta‐catenin signaling and involved in the regulation of neurite outgrowth. Our findings provide new insights into the molecular basis of ODD and inform future genetic studies of oppositional behavior. © 2015 The Authors. *American Journal of Medical Genetics Part B: Neuropsychiatric Genetics* Published by Wiley Periodicals, Inc.

## INTRODUCTION

Oppositional defiant disorder (ODD) shows strong comorbidity with attention‐deficit‐hyperactivity disorder (ADHD), conduct disorder (CD), and mood disorders [Angold et al., 1999], in both epidemiological and clinical samples. To date, the etiological basis of this comorbidity is unclear, although shared genetic influences between these disorders have been postulated to play a role [Faraone et al., 1991; Dick et al., 2005]. Research into ODD has gained momentum due to its relation to later psychopathology such as affective disorders [Copeland et al., 2009] and antisocial personality disorder [Langbehn et al., 1998]. Youths with ADHD frequently show severe impulse control problems and are at high risk for developing ODD. A better understanding of the developmental pathways from ADHD to ODD is crucial to prevent further antisociality and psychopathology. However, there has been little research on the genetics of ODD, perhaps, because this disorder has been viewed primarily as the result of ineffective parenting [Frick et al., 1992]. Nevertheless, the heritability of ODD has been estimated to be around 0.60 [Nadder et al., 1998; Coolidge et al., 2000] and ODD is familial among families of ADHD youth [Petty et al., 2009].

ADHD has been the focus of considerable genetic research. A meta‐analysis of candidate gene studies found several polymorphisms associated with childhood ADHD, including several markers in the dopaminergic and serotonergic systems, and suggested associations in *CHRNA4* and *SNAP25* [Gizer et al., 2009]. Genome‐wide association studies (GWAS) of ADHD did not yet reveal any significant association [Neale et al., 2010; Hinney et al., 2011; Stergiakouli et al., 2012; Ebejer et al., 2013; Yang et al., 2013; Sanchez‐Mora et al., 2015; Zayats et al., 2015]. There is comparatively little work into the molecular genetics of oppositional and disruptive behaviors in children and adolescents. A recent meta‐analysis showed a significant association of the short allele of the polymorphic region (5‐HTTLPR) in the promoter region of the serotonin transporter gene *5‐HTT/SLC6A4* with antisocial behaviors (including aggression) [Ficks and Waldman, 2014], although evidence for this association is conflicting [Vassos et al., 2014]. The short allele has been found to affect negatively the transcription rate of the gene compared to the long allele [Heils et al., 1996], putatively affecting the availability of serotonin in the synaptic cleft and thus increasing the risk for aggressive behavior. Further studies also support the role of dopamine genes in the development of ODD and/or CD. The variable number tandem repeat polymorphism (VNTR) within exon 3 of the dopamine receptor D4 gene (*DRD4)* has been frequently investigated in psychiatric genetic studies and the 7‐repeat allele was found to lead to less efficient dopamine binding and reduced receptor sensitivity. Several studies found individuals with the 7‐repeat allele to have an increased risk for ODD and CD symptoms [Holmes et al., 2002; DiLalla et al., 2009]. In accordance with the findings for *DRD4* and *5‐HTTLPR*, high levels of dopamine and low levels of serotonin were associated with aggression and irritability in humans [Ryding et al., 2008; Duke et al., 2013]. Deregulation of oxytocin (OXT) signaling—for example, as a consequence of genetic variability—also predisposes an individual to antisocial and aggressive behaviors and disrupts prosocial behaviors [Malik et al., 2012]. Two studies found that low levels of OXT are linked to aggressive behaviors in adult males [Fetissov et al., 2006; Lee et al., 2009]. In genetic studies, single nucleotide polymorphisms (SNPs) within the oxytocin receptor gene (*OXTR*) were associated with callous‐unemotional and aggressive behaviors in males and females [Malik et al., 2012; Zai et al., 2012]. To date, seven *OXTR* SNPs (rs1042778, rs6770632, rs237885, rs4564970, rs1488467, rs53576, rs13316193) have been found to be related to aggression, CU behaviors, and/or behavior problems [Park et al., 2010; Campbell et al., 2011; Beitchman et al., 2012; Johansson et al., 2012a; Johansson et al., 2012b; Malik et al., 2012]. Most of the molecular genetic studies of *OXTR* have been limited by small sample sizes, though, and therefore warrant replication.

The phenotypic heterogeneity of ODD complicates the identification of genetic involvement with the occurrence of the disorder. An increasing number of studies supports the need for discrimination of ODD irritable and defiant/vindictive dimensions in community samples of preschoolers, school‐aged children, and adolescents [Stringaris and Goodman, 2009a,2009b; Ezpeleta et al., 2012; Krieger et al., 2013] as well as in children and adolescents referred for ADHD or autism [Aebi et al., 2010; Mandy et al., 2014], which may inform genetic studies. Irritable mood has been suggested to underlie the developmental link between ODD and later affective disorders [Stringaris et al., 2009], and a defiant/vindictive behavioral pattern of ODD is associated with CD and the presence of callous unemotional (CU) traits [Kolko and Pardini, 2010] as well as later criminal outcomes in adulthood [Aebi et al., 2013]. A genetic link between ODD irritable behavior and depression, on the one hand, and between ODD defiant/vindictive aspects and delinquent behavior, on the other, was found in a UK twin sample [Stringaris et al., 2012].

In this study, we aim to investigate the genetic underpinnings of ODD using data from the International Multicentre ADHD Genetics (IMAGE) study [Müller et al., 2011a,2011b] including 750 subjects. We first defined conceptually meaningful dimensions/subtypes of oppositionality in order to improve the power of our analyses by reducing the known heterogeneity of the ODD phenotype [Burke, 2012]. We subsequently tested genetic variants in dopamine, serotonin, and oxytocin signaling pathways for their association with the two dimensions and the two categorical subtypes. We first tested individual polymorphisms earlier found related to such traits, that is, the *DRD4* VNTR 7‐repeat allele, the 5‐HTTLPR short allele, and variants in the *OXTR* gene. In a second step, gene‐wide analysis for *DRD4*, *5‐HTT*, *OXTR*, and gene‐set analysis of the dopamine, serotonin, and oxytocin pathways was performed to test their association with the two dimensions and the two categorical subtypes. Besides adjusting the analyses for age and sex, we also controlled for parental ability to cope with disruptive behavior because parenting behavior has been identified as a major source of ODD [e.g., Burke et al., 2008]. We also tested the interaction between genetic polymorphisms and “parental ability to cope with disruptive behavior” and ODD subtypes/dimensions. In addition to the hypothesis‐driven analyses, we aimed to generate new hypotheses about genetic involvement in ODD. Because genetic overlap as well as differences can be expected to exist between the two dimensions and the two categorical ODD subtypes [Dowell et al., 2010] and to maximize power of our analyses [Galesloot et al., 2014], we used a multivariate genome‐wide association testing framework. Employing bioinformatics and literature mining, we integrated top‐ranked findings from the GWAS into a landscape of proteins and molecules that regulate biological signaling cascades, providing important new insights into the genetic etiology of ODD.

## MATERIALS AND METHODS

### Sample

The present study is based on 750 probands from the International Multicentre ADHD Genetics (IMAGE) study. Participants of the IMAGE study were European Caucasians aged 5–17 years, who had been recruited in 12 child and adolescent psychiatry clinics representing eight countries: Belgium, Germany, Switzerland, Holland, Ireland, Israel, Spain, and the United Kingdom. Approval was obtained by the Institutional Review Board of SUNY Upstate Medical University and from ethical review boards within each country. A detailed description of the study design and assessment procedures has been provided in previous publications [Müller et al., 2011a,2011b]. In short, entry criteria for probands were a clinical diagnosis of ADHD based on DSM‐IV criteria and access to one or both biological parents and one or more full siblings for DNA collection and clinical assessment. Exclusion criteria applying to both probands and siblings included autism, epilepsy, IQ <70, brain disorders, and any genetic or medical disorder associated with externalizing behaviors that might mimic ADHD. The full sample of the IMAGE project amounts to 1,067 subjects. Out of this sample with ADHD combined type, 774 subjects with full information on ODD phenotypes and covariates (see below) were included in the analyses. Genome‐wide imputed genotypes (HAPMAP2) and variable number of tandem repeats (VNTR) were available for 750 subjects. Attrition analyses showed that the 317 subjects, who were not included in the analyses, did not differ from the participating 750 subjects in terms of sex (male sex 86.8% vs. 87.7%; χ^2^ = 0.20, df = 1, *P* *= n.s*.), age (10.94 vs. 10.67 years; t = 1.43, df = 1,065, *P* *=* *n.s*.), and ODD diagnosis (69.0% vs. 64.1%; χ^2^ = 2.32, df = 1, *P* = *n.s*.).

### Measures

The long form of the revised Conners parent rating scale (CPRS‐R:L) was used in the present study [Conners, 1997; Conners et al., 1998]. Subtypes and dimensions of oppositionality were assessed by use of the 10 items (0 = not true, 1 = little true, 2 = much true, 3 = very much true) of the CPRS‐R:L oppositional scale. In total, four different phenotype (two dimensional and two categorical) measures were included in the present study and tested for differences in the candidate‐based and hypothesis‐free analyses (see below). The use of dimensional as well as categorical measures of ODD is in line with previous research confirming (1) separate but correlated dimensions of ODD [Stringaris and Goodman, 2009a,2009b; Aebi et al., 2010; Ezpeleta et al., 2012; Aebi et al., 2013; Krieger et al., 2013] and (2) distinct subtypes of irritable and severe forms of ODD [Burke, 2012; Kuny et al., 2013; Althoff et al., 2014].

**Figure 1:**
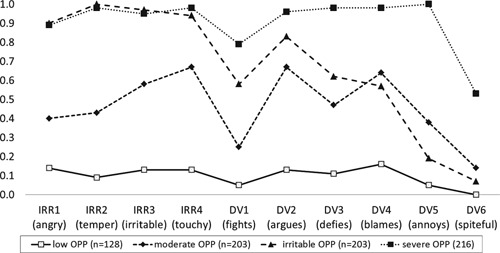
Mean scores of dichotomized items of the Conners parent scale (CPRS‐R:L) oppositional scale assessing irritable (IRR1‐IRR4), and defiant/vindictive (DV1–DV6) behaviors as a function of latent classes for children and adolescents with ADHD combined type (N = 750). OPP, oppositionality.

1. Two dimensions were defined on theoretical grounds, which reflected the two previously described dimensions of ODD [Stringaris et al., 2012; Aebi et al., 2013], namely defiant/vindictive (P1) and irritable (P2). The items related to P1 with scores ranging from 0 to 18 and P2 with scores from 0 to 12 for P2 are shown in Figure 1. Internal consistencies (Cronbach alpha) amounted to 0.79 and 0.82 for the defiant/vindictive and the irritable dimension, respectively. Because of a right skewed distribution, a Blom transformation [Blom, 1958] of P2 was performed.
2. Two further dichotomous subtypes were based on findings from a latent class analysis (LCA). LCA was performed using poLCA package [Linzer and Jeffrey, 2011] in R statistic software [R Development Core Team, 2011]. All of the 10 dichotomized CPRS‐oppositionality items (0 and 1 were scored as absent; 2 and 3 were scored as present) were included in analysis. One to five class models were compared, and the Bayesian Information Criterion (BIC) and the Akaike Information Criterion (AIC) were used to determine the number of classes. The four class solution, which fitted the data best (BIC = 11,169; AIC = 10,955), contained classes labeled *low oppositionality (OPP), moderate OPP, irritable OPP*, and *severe OPP* (see Fig. 1). Because of our interest in severe forms of ODD, we defined the following dichotomous phenotypes: a dichotomous subtype P3, with 0 representing “low OPP/moderate OPP” (n = 331) and 1 representing “irritable OPP/severe OPP” (n = 419), and a dichotomous subtype P4, with 0 representing “low OPP/moderate OPP/irritable OPP” (n = 534) and 1 representing “severe OPP” (n = 216).

The DSM‐IV diagnoses of ODD/CD and parental ability to cope with disruptive behaviors was coded from the diagnostic interview (parental account of childhood symptoms [PACS]; Chen and Taylor, 2006; Taylor et al., 1986] A parent (usually the mother) responded to a 7‐point Likert‐scale ranging from 0 (efficient coping) to 7 (abusive parental behavior) measuring maternal and paternal coping with disruptive behaviors. A mean score was used when information for both parents was available. Furthermore, the oppositional scale of the Conners' teacher rating scale (CTRS‐ R:L; [Conners, 1997]) and the conduct problem scale of the Strengths and Difficulties Questionnaire [Goodman, 1997] were used for phenotype description.

### DNA Collection and Genotype Assays

Sample collection and DNA isolation has been described previously [Brookes et al., 2006]. Genome‐wide genotyping and data cleaning was performed as part of the GAIN study using the Perlegen 600K genotyping platform, as described in Neale et al. [2008]. To increase genomic coverage, imputation was performed using MACH and the Hapmap 2 (Release 22 Build 36) reference data set [Y. Li et al., 2010]. Quality control was performed on the imputed data, and SNPs with imputation quality scores lower than 0.30, a minor allele frequency lower than 0.01, and those failing the Hardy–Weinberg equilibrium test at a threshold of *P* ≤ 10^−5^ were excluded. In addition, SNPs and subjects with missingness rates higher than 0.05 were removed from the data. Distributed over 22 autosomes, 1,871,025 SNPs were left for analysis.

Genotyping of candidate polymorphisms (DRD4 exon 3 VNTR; 5‐HTTLPR) was performed at the *SGDP* laboratories in London or at the Human Genetics department of the Radboudumc in Nijmegen, the Netherlands. Standard PCR protocols were used, as previously described [Brookes et al., 2006; Thissen et al., 2015].

### Statistical Analyses

#### Analysis of candidate polymorphisms

For the oxytocin receptor gene *OXTR*, only one of the seven SNPs previously linked with aggression was present in the data (rs1488467). Linear and logistic regression analyses were used to test the effects of the *DRD4* exon 3 variant (presence/absence of the 7‐repeat allele: 7R/7R and 7R/other vs. other/other), the *5‐HTT* variant (presence/absence of the 5‐HTTLPR short allele: S/S and S/L vs. L/L), and the *OXTR* rs1488467 SNP (presence/absence of C: C/C and C/G vs. G/G) on the ODD dimensions/subtypes. Variables included in the model were age, sex, and parental ability to cope with disruptive behaviors, as well as the interaction of *DRD4*, 5‐HTTLPR, and OXTR rs1488467 genotype with parental ability to cope with disruptive behaviors. In addition, since only one of the seven *OXTR* SNPs of interest was present in the data, outcome of the association analysis of all SNPs located in that region was plotted to find out if an association signal was presented by closely related linked SNPs.

#### Gene‐wide and gene‐set analyses

Gene‐wide analysis was applied for *5‐HTT* as well as for *DRD4* and *OXTR* using a mass‐univariate approach, to take potential allelic heterogeneity into account and test if a combination of SNPs located in these genes showed association with the ODD dimensions/subtypes. Similarly, gene‐set analysis was performed for all genes involved in serotonin, dopamine, and oxytocin neurotransmission. A list of genes included in each pathway‐wide analysis can be found in Supplementary Table SI. All available variants of each gene were extracted, including variants within a 100 kilobase (kb) flanking region of each gene to capture regulatory sequences. The effect of common variants of each gene or gene‐set of interest on the two dimensions and the two categorical subtypes was investigated using the statistical approach described by Bralten et al. [2013] consisting of SNP‐by‐SNP regression and estimation of the effect of the whole gene or gene‐set. For both gene‐wide and gene‐set based analyses, linkage disequilibrium‐pruned genotyping data were prepared, using the “indep” command in Plink [Purcell et al., 2007] with a r^2^ threshold of 0.8.

#### Correction for multiple testing

Results were considered to be significant if they reached the Bonferroni corrected *P*‐value threshold for multiple testing (0.05 divided by the number of phenotypes, polymorphisms, and gene(‐sets) tested; *P*‐value threshold = 1.4E‐3).

#### Multivariate genome‐wide association study

We performed a multivariate GWAS to capture covariance among the different correlated ODD dimensions/subtypes and to increase the power for finding genetic associations. Using only a single test for association instead of four has the additional advantage of a reduced multiple testing burden. Following analysis of correlation between traits, we assessed association between genetic markers and the two dimensions and the two categorical subtypes using the MQFAM multivariate extension of PLINK [Ferreira and Purcell, 2009]. Residuals obtained for each subtype after adjustment for age, sex, parental ability to cope with disruptive behavior, and four population components derived from multidimensional scaling analysis were used as input. The MQFAM method uses canonical correlation analysis to identify the linear combination of traits that maximizes the covariance between a marker and the traits. It can be used for analysis of a combination of quantitative and binary traits [Ferreira and Purcell, 2009; Galesloot et al., 2014]. For each SNP included in the analysis, a loading is calculated in the output which reflects the contribution of each phenotype to the association results. Top‐SNPs (*P* < 1.00E‐5) from the multivariate GWAS were investigated for their location in or around genes and for their performance in univariate analysis, which provided information on the direction of effect.

#### Molecular landscape building: bioinformatics and literature analyses

To increase the understanding of the molecular basis of ODD, we aimed at integrating the top findings from the GWAS into a landscape of functionally interacting proteins and molecules that regulate biological signaling cascades. First, a list of independent association regions was obtained by clumping the results using PLINK [Purcell et al., 2007]. SNPs in LD (r^2^≥ 0.2) within 10,000 kb of a more significant index SNP were discarded. Second, a threshold of *P* < 1.00E‐04 was applied for index SNPs, resulting in 65 LD‐independent regions. The chosen statistical cut‐off for association of *P* < 1.00E‐04 is often used to designate ‘suggestive’ association and has been previously used in studies of neurodevelopmental disorders (ADHD and autism) [Poelmans et al., 2013, 2011b]. Third, a list of top genes was compiled. Gene annotation was performed when an index SNP was located within an exon, an intron, or untranslated region of the gene, or when an index SNP was located within a region 100 kb downstream or upstream of the gene to capture regulatory sequences [Veyrieras et al., 2008; Gherman et al., 2009; Nicolae et al., 2010; Pickrell et al., 2010].

We then conducted a canonical pathway analysis of the list of top‐ranked genes from the multivariate GWAS, using the Ingenuity software package (http://www.ingenuity.com). For this pathway enrichment analysis, Ingenuity draws on the Ingenuity Knowledge Base which is based on information from published literature as well as on various other sources including gene expression and gene annotation databases. An enrichment *P*‐value is calculated for each pathway with the right‐tailed Fisher's exact test and correction for multiple testing is performed using the Benjamini–Hochberg correction. Subsequently, we searched the literature for the function of the proteins encoded by all the top‐ranked genes from the multivariate GWAS, using UniProtKb (http://www.uniprot.org/uniprot) and Pubmed (http://www.ncbi.nlm.nih.gov/). The landscape building approach described here has been used in earlier studies of neurodevelopmental disorders [Poelmans et al., 2011a,^2013^, 2011b] Lastly, the genes from the list with top findings were investigated for previous implication in the etiology of neurodevelopmental or neuropsychiatric disorders using Ensembl release 75 [Flicek et al., 2014] and the NCBI databases (http://www.ncbi.nlm.nih.gov/).

## RESULTS

### Descriptives

The final sample (N = 750) consisted of 658 boys (87.7%) and 92 girls (12.3%) aged 5–18 years (mean = 10.67 years, SD = 2.77). According to the PACS interview, 481 (64.1%) children and adolescents fulfilled DSM‐IV criteria for ODD and 170 (22.7%) for CD. Bivariate correlations of the two dimensions and the two categorical subtypes are shown in Supplementary Table SII. All correlations were significant and moderate. Furthermore, all dimensions/subtypes were slightly correlated to teacher ratings of oppositionality (CTRS), and moderately correlated to SDQ conduct problems and DSM‐IV diagnosis of ODD / CD (also shown in Supplementary Table SII).

### Candidate Polymorphisms

No associations of *DRD4*, 5‐HTTLPR, and *OXTR* rs1488467 were observed for any of the four measures, nor were any interactions of parental ability to cope with disruptive behaviors with these genotypes observed (Table I). Parental ability to cope with the child's disruptive behaviors was significantly associated with all four ODD measures (except for 5‐HTTLPR analysis of severe oppositionality (P4)). Age was positively associated with irritability (P2) and irritable/severe oppositionality (P3), in all three models. There appeared to be no SNPs closely located to, and in high linkage disequilibrium with, *OXTR* SNP rs1488467 that show association with the ODD dimensions/subtypes (Supplementary Fig. S1).

**Table I:** Linear and Logistic Regressions of the *DRD4* Genotype (Presence/Absence of the Seven Repeat Allele: 7R7R and 7R/Other Vs. Other/Other), of the *HTTLPR* Genotype (Presence/Absence of the Short Allele: S/S and S/L Vs. L/L), and of the *OXTR* Genotype rs1488467 (Presence/Absence of C: C/C and C/G Vs. G/G) Predicting the Four Phenotypes of ODD

Phenotypes	P1	P2 (transformed)	P3	P4
Variables	Β	Β	Β	Β
*DRD4* genotype				
*DRD4* (7R7R and 7R/other vs. other/other)	−0.39 n.s.	−0.08 n.s.	0.00 n.s.	−0.10 n.s.
Parent coping (centered)	0.65^***^	0.16^***^	0.28^***^	0.27^***^
*DRD4* (7R7R and 7R/other vs. other/other) × parent coping (centered)	0.02 n.s.	−0.08 n.s.	−0.10 n.s.	−0.12 n.s.
Sex (0 = female, 1 = male)	0.69 n.s.	0.19 n.s.	0.33 n.s.	0.35 n.s.
Age	0.09 n.s.	0.04^**^	0.07^*^	0.05 n.s.
HTTLPR genotype				
5‐HTTLPR (S/S and S/L vs. L/L)	0.51 n.s.	−0.02 n.s.	−0.03 n.s.	0.20 n.s.
Parent coping (centered)	0.73^**^	0.16^**^	0.25^*^	0.23 n.s.
5‐HTTLPR (S/S and S/L vs. L/L) × parent coping (centered)	−0.13 n.s.	−0.04 n.s.	0.00 n.s.	−0.02 n.s.
Sex (0 = female, 1 = male)	0.64 n.s.	0.19 n.s.	0.34 n.s.	0.28 n.s.
Age	0.08 n.s.	0.04^**^	0.07^*^	0.04 n.s.
*OXTR* rs1488467 genotype				
rs1488467 (C/C and C/G vs. G/G)	0.14 n.s.	0.12 n.s.	0.13 n.s.	−0.08 n.s.
Parent coping (centered)	0.63^***^	0.13^***^	0.25^***^	0.24^***^
rs1488467 (C/C and C/G vs. G/G) × parent coping (centered)	0.35 n.s.	0.10 n.s.	0.11 n.s.	0.02 n.s.
Sex (0 = female, 1 = male)	0.73 n.s.	0.22^*^	0.35 n.s.	0.31 n.s.
Age	0.08 n.s.	0.03^*^	0.06^*^	0.04 n.s.

Note: P1, defiant vindictive dimension; P2, irritable dimension; P3, irritable/severe oppositionality; P4, severe oppositionality.

^*^ Significance (two sided), *P* < 0.05

^**^ Significance (two sided), *P* < 0.01

^***^ Significance (two sided), *P* < .001.

### Gene‐Wide and Gene‐Set analyses

Findings for the *5‐HTT*, *DRD4*, and *OXTR* genes and the neurotransmission pathways are shown in Table II. None of the analyses revealed a significant association with any of the four ODD phenotypes.

**Table II:** *P*‐Values of Gene‐Wide and Gene‐Set‐Based Analysis of *5‐HTT, DRD4*, and *OXTR* Genes and the Neurotransmission Pathways for Serotonin, Dopamine, and Oxytocin

	Gene‐wide analysis			Gene‐set analysis		
Phenotype	*5‐HTT* (20 SNPs)	*DRD4* (14 SNPs)	*OXTR* (71 SNPs)	Serotonin (942 SNPs)	Dopamine (2568 SNPs)	Oxytocin (360 SNPs)
P1	0.2508	0.2756	0.3101	0.3458	0.5612	0.6798
P2	0.6463	0.9455	0.5737	0.5493	0.4726	0.9272
P3	0.9445	0.3128	0.9649	0.515	0.276	0.9991
P4	0.1632	0.7257	0.5579	0.5012	0.274	0.9377

Note: P1, defiant vindictive dimension; P2, irritable dimension; P3, irritable/severe oppositionality; P4, severe oppositionality.

### Multivariate Genome‐Wide Association Study

**Figure 2:**
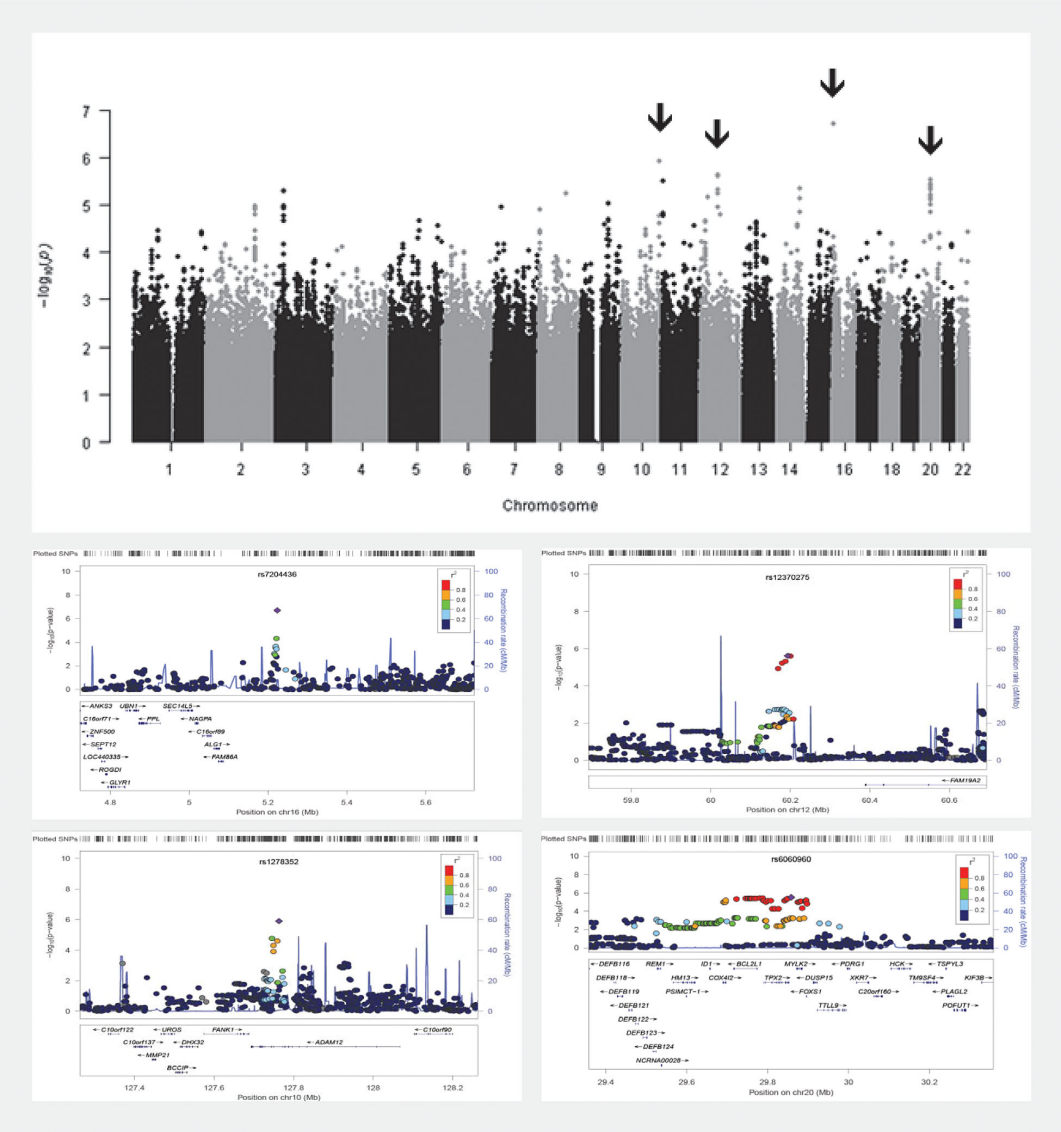
**Top:** Manhattan plot of multivariate GWAS including ODD subtypes P1 (defiant vindictive), P2 (irritable), P3 (0 representing “low OPP/moderate OPP” and 1 representing “irritability/severe OPP”), and P4 (0 representing “low OPP/moderate OPP/irritability” and 1 representing “severe OPP”). **Bottom:** Top four regions (indicated by arrows in the manhattan plot) containing SNPs showing association at *P* < 1.00E‐5 in the multivariate GWAS. Top SNPs for each region are depicted in purple; rs7204436 on chromosome 16 (*P* = 1.98E‐07), rs1278352 on chromosome 10 (*P* = 1.24E‐06), rs12370275 on chromosome 12 (*P* = 2.41E‐06), and rs6060960 on chromosome 20 (*P* = 3.00E‐06). OPP, oppositionality.

As expected given the modest sample size (n = 750), multivariate GWAS did not result in genome‐wide significant findings (*P* < 5.0E‐08; [Dudbridge and Gusnanto, 2008]) (see Fig. 2, and Supplementary Fig. S2 for the Quantile–Quantile plot). Supplementary Table SIII presents the 53 SNPs showing association with the ODD dimensions and subtypes at *P* < 1.00E‐5, together with their respective loadings reflecting the contribution of each phenotype to the association results and their performance in univariate analysis. The top three findings were for rs7204436 (*P* = 1.98E‐07) located in an intergenic region on chromosome 16, rs1278352 (*P* = 1.24E‐06) located in an intronic region of the *ADAM12* gene on chromosome 10, and rs12370275 (*P* = 2.41E‐06) located in an intergenic region on chromosome 12 (Fig. 2). Also of interest is a region on chromosome 20 with a large number of SNPs in high LD showing a strong association signal. This region is located on chromosome 20q11.21 and is spanning several genes (*COX4I2, BCL2L1, TPX2, MYLK2, FOXS1, TTLL9*) (also depicted in Fig. 2).

### Molecular Landscape Building

**Figure 3:**
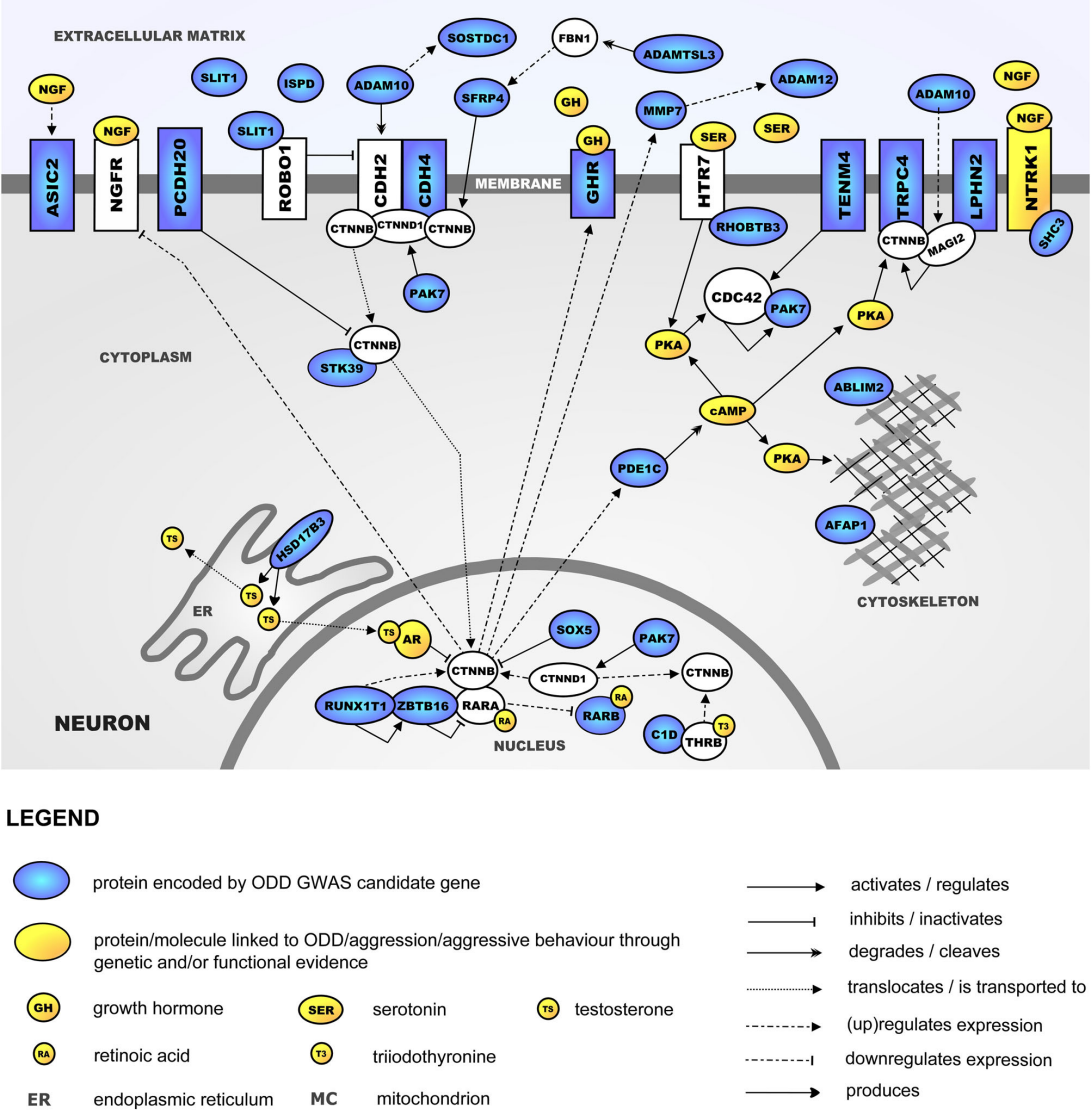
Neurite outgrowth‐regulating molecular landscape implicated in ODD. The evidence linking the molecules in the landscape to neurite outgrowth can be found in the Supplementary Information.

Using the criteria as described in the methods section, gene annotation was performed for 44 out of 65 independent SNPs with a *P* < 1.00E‐04, resulting in a list of 53 top‐ranked genes (Supplementary Table SIV). The bioinformatics analysis with Ingenuity revealed significant enrichment of the canonical pathways “Inhibition of matrix metalloproteases” (P_corrected_ = 1.19E‐2), “Axonal guidance signaling” (P_corrected_ = 2.60E‐02), and “Wnt/Beta‐catenin signaling” (P_corrected_ = 2.60E‐02), with the proteins encoded by nine of the top‐ranked genes belonging to one or more of these pathways (Table III). Importantly, all proteins encoded by these nine genes play a role in neurite outgrowth. In addition, the subsequent literature analysis revealed that in total, 28 of the 53 top‐ranked ODD genes (53%) interact in a molecular landscape centered around β‐catenin signaling and involved in regulating neurite outgrowth (depicted in Fig. 3). This landscape encompasses signaling cascades that are important for the neural modulations necessary for the growth of axons in a specific direction. The evidence linking the molecules in the landscape to neurite outgrowth is described in detail in the Supplementary Information.

**Table III:** Three Canonical Pathways That Were Significantly Enriched in the Top 53 ODD GWAS Genes, Using Ingenuity Pathway Analysis (www.ingenuity.com)

Canonical pathway	Genes	Significance^*^	Adjusted significance^**^
Inhibition of matrix metalloproteases	***ADAM10, ADAM12, MMP7***	1.20E‐04	1.19E‐02
Axonal guidance signaling	***ABLIM2, ADAM10, ADAM12, MMP7, PAK7, SLIT1***	6.46E‐04	2.60E‐02
Wnt/*β*‐catenin signaling	***MMP7, RARB, SFRP4, SOX5***	7.86E‐04	2.60E‐02

The genes encoding proteins that could be directly placed in the odd landscape are indicated in bold

^*^ Single test *P*‐value calculated with the right‐tailed Fisher's exact test and taking into consideration both the total number of molecules from the analysed dataset and the total number of molecules that is linked to the same gene category according to the Ingenuity Knowledge Base.

^**^ Multiple test‐corrected *P*‐values using the Benjamini–Hochberg correction (*P* < 0.05).

Fifteen of the top‐ranked genes have also been implicated previously in the etiology of neurodevelopmental and/or neuropsychiatric disorders. A summary of these genes and previous findings from literature can be found in Supplementary Table SV.

## DISCUSSION

The aim of this study was to reduce the known heterogeneity in the ODD phenotype in order to improve the power to detect the genetic underpinnings. We first identified four conceptually meaningful subtypes and dimensions of oppositionality in the IMAGE sample. We then tested specific polymorphisms and genes/gene‐sets that have been previously implicated in aggression/disruptive behavior for their effect on the two dimensions and the two categorical subtypes. In addition to these hypothesis‐driven analyses, we aimed to generate new hypotheses about genetic involvement in ODD by performing multivariate GWAS. By using bioinformatics analysis and literature mining, we found that top findings obtained from the GWAS fit into a neurite outgrowth‐regulating molecular landscape.

Previous research has focused on various dimensions within oppositional defiant behaviors [Stringaris and Goodman, 2009b; Aebi et al., 2010]. Further studies have attempted to identify discrete classes of children and adolescents according to their oppositional behavior profiles. Consistent with previous research [Kuny et al., 2013; Althoff et al., 2014], LCA in the present study revealed a low symptom endorsement type, an irritable type, and a severe type with elevated scores on all symptoms. In contrast to these previous findings, we additionally found a moderate oppositional type with intermediate scores on all symptoms, but not a specific defiant/vindictive type. Considering the large sample size and the multi‐site data collection for the sample of the present study [Müller et al., 2011a,2011b] one may conclude that, most probably, children with ADHD more often show the full range of ODD symptoms rather than defiant/vindictive symptoms only. In contrast, irritability symptoms are frequently co‐occurring in ADHD children and may represent a specific subtype of ADHD [Fernandez de la Cruz et al., 2015].

Although we tried to reduce the heterogeneity of ODD by identifying conceptually meaningful subtypes and dimensions of oppositionality, we did not observe any significant associations or interactions with previously postulated candidates (SNPs, genes, and pathways). This is not surprising in light of inconsistent reports of *DRD4*, *5‐HTT*, and *OXTR* effects on externalizing behaviors (e.g., [Kirley et al., 2004; Beitchman et al., 2012; Malik et al., 2012; Lavigne et al., 2013]), and the small effect sizes of most genetic risk factors for behavioral measures. A recent meta‐analysis did not confirm a relation of *DRD4* exon3 and 5‐HTTLPR to aggression and violence [Vassos et al., 2014]. Furthermore, our findings mirror those of a previous study that did not find a *DRD4*/5‐HTTLPR‐ interaction with parental support for ODD in 4‐year‐old children [Lavigne et al., 2013]. Parenting behavior was moderately to strongly associated with the defined ODD dimensions and subtypes. In line with behavioral theories on negative parent‐child interactions (e.g., coercive behaviors; [Patterson, 1982]), parenting behavior was most strongly associated with defiant/vindictive behaviors. Since parental ability to cope with the child's disruptive behavior was rated by PACS interviewers, and symptoms of oppositionality were rated by parents, confounding of these variables by rater‐effects is unlikely.

In order to obtain new insights into genetic risk factors for ODD that can inform future investigations of the neurobiology related to oppositional behavior, we also conducted a multivariate GWAS using the four ODD subtypes and dimensions. We found 53 markers that showed association with at least one of the four phenotypes at *P* < 1.00E‐5. The strongest association with oppositional behavior was found for rs7204436 (*P* = 1.98E‐07) located in an intergenic region on chromosome 16. Although no genes are located nearby, a novel microRNA was found 30 kb from the marker which might regulate genes involved in the etiology of oppositional behavior.

Out of 53 markers with *P* < 1.00E‐05, 46 were located in a region on chromosome 20q11.21 spanning the genes *COX4I2, BCL2L1, TPX2, MYLK2, FOXS1*, and *TTLL9*. It can be hypothesized that of these genes, *BCL2L1* is the most likely candidate causing suggestive association of the region with oppositional behavior. The long isoform Bcl‐S(L) is an anti‐apoptotic regulator expressed at high levels in both the developing and the adult brain [Krajewska et al., 2002]. Interestingly, it regulates neurotransmitter release and retrieval of vesicles in neurons, thereby influencing presynaptic plasticity [Li et al., 2013]. Recently, it has also been shown that *BCL2L1* is associated with volume of the putamen in a GWAS of subcortical volumes in 30,717 individuals from 50 cohorts [Hibar et al., 2015]. *BCL2L1* is not present in our top gene list because of filtering during the clumping procedure.

Genome‐wide studies of aggression phenotypes are starting to emerge. A GWAS of CD had been performed before in the current ADHD sample [Anney et al., 2008], where one of the three phenotypes used was defined as the sum score for 12 CPRS‐R:L items, giving perhaps a better representation of ODD than CD. In contrast, we assumed in the present study that combining biologically valid and less heterogeneous subtypes of ODD through a multivariate approach would improve power to define new hypotheses about the genetics of ODD. The top SNPs reported by Anney et al. [2008], who performed family‐based transmission disequilibrium tests (TDT), did not reach suggestive significance (*P* < 1.00E‐04) in our study (Supplementary Table SVI). A few other GWAS of aggression‐related phenotypes have been reported to date. We compared our association results for the oppositional phenotypes to the top results of four published aggression related genome‐wide association studies [Alliey‐Rodriguez et al., 2011; Dick et al., 2011; Tielbeek et al., 2012; Mick et al., 2014]. None of the SNPs in a 100 kb region surrounding these reported top results reached the threshold for suggestive association in our study (*P* < 1.00E‐4) (Supplementary Fig. S3). Interestingly though among our list of top genes is *EPDR1* (ependymin related 1). Ependymin is involved in control of aggressive behavior in fish, where it is a neurotrophic factor that plays a role in neuronal regeneration and adhesion [Sneddon et al., 2011]. The mammalian ependymin related protein 1 shows significant sequence similarity to piscine ependymins and has been proposed to be the human homolog of the piscine ependymin [Nimmrich et al., 2001]. These findings make *EPDR1* an interesting candidate gene for future investigations of genetic contributions to aggression phenotypes. An additional comparison of SNPs reaching suggestive association in our study (*P* < 1.00E‐4) with a list of ADHD GWAS top hits with *P*‐value <1.00E‐05 (Supplementary Table SVII, adapted from Zayats et al. [2015]), did not reveal overlap of our findings with top hits from genome‐wide studies of ADHD phenotypes.

As could be expected based on sample size, our multivariate approach did not retrieve any region that yielded genome wide significant association with ODD. Nevertheless, using the described landscape building approach, we have integrated the top‐ranked findings of the GWAS into a molecular landscape involved in regulating neurite outgrowth. More than half of our top‐ranked ODD genes were found to interact functionally within this landscape, identifying neurite outgrowth as a biological process that is important for the etiology of ODD. This is in line with neuroimaging studies indicating that aggressive behavior is associated with dysfunctional brain circuitry involved in emotion regulation and decision making [Blair, 2013]. Moreover, current models of aggression postulate an impaired structural and functional connectivity between prefrontal areas and subcortical structures such as the amygdala [Rusch et al., 2007; Siever, 2008; Saxena et al., 2012]. Indeed, alterations in the efficiency or direction of neurite outgrowth may underlie these dysfunctions.

The identified molecular landscape centers around Beta‐catenin (CTNNB) signaling. CTNNB has a pivotal function in an important signaling cascade leading to neurite outgrowth. The process of neurite outgrowth can be initiated at the neuronal cell membrane, where the binding of ligands from the extracellular matrix to their receptors leads to the modulation of downstream molecular cascades in the cytoplasm, cytoskeleton, and nucleus that are involved in regulating neurite outgrowth. Importantly, several proteins and signaling molecules in the landscape (highlighted in yellow in Fig. 3)—including serotonin, testosterone, triiodothyronine, growth hormone, and retinoic acid—have been associated with ODD or aggressive behavior through genetic or functional evidence (Supplementary Table SVIII). Starting with the findings on genetic deficits in the metabolism of neurotransmitters in aggressive patients [e.g., Valzelli, 1981] and the discovery of a nonsense mutation in the *MAOA* gene leading to a syndrome characterized by violent behavior [Brunner et al., 1993], the key role of monoamines and especially serotonin in aggression has been demonstrated in a wide variety of human and animal studies [Anholt and Mackay, 2012]. Several studies also show a correlation of levels of the male hormone testosterone and aggression [Pavlov et al., 2012] and it has been proposed that an altered testosterone‐to‐cortisol ratio may be associated with aggression in humans [Haller, 2012; Montoya et al., 2012]. Further, thyroid hormones are associated with stress, and elevated levels of the active thyroid hormone triiodothyronine (T3) are associated with conduct disorder and criminal behavior [Ramklint et al., 2001; Stalenheim, 2004]. In addition, several animal studies suggest that growth hormone (GH) influences aggressive behavior. For example, *GHRH* knock‐out mice with GH deficiency show reduced aggressive behavior which can be normalized by GH replacement [Sagazio et al., 2011]. Lastly, chronic administration of synthetic retinoic acid to rats reduced aggression‐ and increased flight‐related behaviors in the resident‐intruder paradigm [Trent et al., 2009]. The fact that these and other molecules active within our landscape have been associated previously with aggressive behavior provides corroborating evidence for the involvement of neurite outgrowth in aggression etiology.

Of note, alterations in neurite outgrowth are not specific to the etiology of ODD, as neurite outgrowth has also been shown to play a role in the pathogenesis of other neurodevelopmental disorders such as ADHD, autism spectrum disorders (ASD), dyslexia, and schizophrenia [Penzes et al., 2011; Poelmans et al., 2011a,^2013^, 2011b]. It has been hypothesized in these studies that each of these disorders may in part be explained by different functional consequences and different primarily affected brain regions of disturbed neurite outgrowth. Psychiatric disorders, including ODD, are currently classified based on clinical presentation rather than underlying etiology. Hence, shared genetic etiology can be expected to exist not only between definable subtypes of psychiatric disorders, but also between different psychiatric disorders as currently classified in clinical practice. This notion is also supported by a recent study [Cross‐Disorder Group of the Psychiatric Genomics et al., 2013) that detected substantial genetic correlations between five major psychiatric disorders and by the fact that 15 out of the 53 top ranked genes of our study have previously been associated with neuropsychiatric and neurodevelopmental disorders.

This study is based on a representative clinical sample from eight European countries. Psychometrically reliable and valid measures and methods (e.g., LCA) were used for phenotype definitions and advanced methods were performed in gene‐set and genome‐wide analyses. However, the present study is limited to data obtained from children and adolescents with ADHD combined type (which is often comorbid with ODD) and although our findings may not be generalized to other clinical and community samples, the overlap of our top findings with results in other genetic studies of psychiatric disorders suggests a broader validity. Our results were based on Caucasian subjects only and the sample consisted mostly of male subjects. Due to missing information in the PACS and other instruments, our sample was reduced to 750 probands. However, attrition analyses did not show significant differences between probands included in the sample and drop‐outs.

A potential source of bias in our bioinformatics analysis arises from the fact that brain‐expressed genes are relatively large. Therefore, brain‐expressed genes may be over‐represented in our GWAS results. If large genes are more likely found to be associated by chance (because they contain more SNPs), this should be the case in GWASs of both psychiatric disorders and non‐psychiatric disorders that do not originate in the brain. However, previous studies have compared enrichment results for psychiatric disorders with results from Crohn's disease and diabetes mellitus [Poelmans et al., 2013, 2011b] and showed that the “neurological disease” category enriched in the psychiatric GWASs showed very little or no enrichment in Crohn's disease or diabetes. Combined with the fact that 53% of our ODD top genes also fitted in the molecular landscape for neurite outgrowth based on extensive literature mining, we argue that although some genes in the landscape may have been chance findings, most candidate genes from the GWAS represent true findings contributing to our phenotype. Future studies conducting pathway analyses using algorithms that address potential confounders such as the large size of brain genes will be of additional information [Holmans et al., 2009; Lee et al., 2012].

In summary, the present findings confirmed the existence of various subgroups of youths with different oppositional symptom profiles. However, against our expectations the examined ODD dimensions and subtypes were not associated with previously described candidate genes and pathways. By employing a multivariate genome‐wide association approach, we identified several genetic susceptibility loci that may inform future theories on the etiology of oppositional behavior. We also identified a biological landscape of molecular signaling cascades involved in neurite outgrowth providing new insights into the etiology of ODD. In part, our findings may reflect shared genetic risk factors for psychiatric disorders. We hope to encourage further investigations toward a biologically informed classification of psychopathology.

## CONFLICTS OF INTEREST

M.A., Mv.D., G.P., and Kv.H. have no conflicts of interest to disclose.

J.B. has been in the past 3 years a consultant to/member of advisory board of/and/or speaker for Janssen Cilag BV, Eli Lilly, and Servier. He is not an employee of any of these companies, and not a stock shareholder of any of these companies. He has no other financial or material support, including expert testimony, patents, royalties.

E.S.B. declares the following competing interests during the 3 years prior to September 2014: Fees for speaking, consultancy, research funding, and conference support from Shire Pharma: Speaker fees from Janssen Cilag, Medice & Qbtech: Book royalties from OUP and Jessica Kingsley: Consultancy from Neurotech solutions.

A.S. receives Royalties from Cambridge University Press for the Maudsley Reader in Phenomenological Psychiatry.

S.V.F. has received in the past year consulting income, travel expenses and/or research support from Akili Interactive Labs, Alcobra, VAYA Pharma, and SynapDx and research support from the National Institutes of Health (NIH). His institution is seeking a patent for the use of sodium–hydrogen exchange inhibitors in the treatment of ADHD. In previous years, he received consulting fees or was on Advisory Boards or participated in continuing medical education programs sponsored by: Shire, Alcobra, Otsuka, McNeil, Janssen, Novartis, Pfizer, and Eli Lilly.

B.F. Barbara Franke has received a speaker fee from Merck.

H.C.S. has been in the last 36 months a member of the advisory board and/or received payment for lectures including service on speakers bureaus by Shire and Medice. He receives book royalties from Cambridge University Press, Elsevier, Hogrefe, and Kohlhammer publishers. The present work is unrelated to the above grants and relationships.

## Supporting information

Additional supporting information may be found in the online version of this article at the publisher's web‐site.

**Table SI**. Lists of genes included in each of the gene‐set analyses.
**Table SII**. Bivariate correlations of ODD subtypes.
**Table SIII**. Top SNPs with p < 1.00E‐05 for association in the multivariate GWAS and their performance in univariate analysis.
**Table SIV**. Index SNPs showing association with ODD at p < 1.00E‐04 after clumping of multivariate association results.
**Table SV**. Top‐ranked genes previously implicated in the etiology of neurodevelopmental or neuropsychiatric disorders.
**Table SVI**. SNPs showing association signal P<1.00E‐5 in the GWAS by Anney et al. (Anney et al., 2008) and their performance in our multivariate GWAS.
**Table SVII**. List of ADHD GWAS top hits (p‐value < 1.00E‐05) that were compared with top hits from our study.
**Table SVIII**. Genes/proteins/molecules from the molecular landscape linked to aggressive behavior through genetic and/or functional evidence.
**Figure S1**. Outcome of the association analysis of the two ODD subtypes and the two ODD dimensions for all SNPs located in the OXTR region.
**Figure S2**. Quantile quantile plot for the multivariate GWAS.
**Figure S3**. Comparison with results of previous aggression related GWAS.
**Figure S4**. Comparison with results of previous aggression related GWAS.
