# Supplementary material for: Gene‐set and multivariate genome‐wide association analysis of oppositional defiant behavior subtypes in attention‐deficit/hyperactivity disorder
Source: Am J Med Genet B Neuropsychiatr Genet. 2015 Jul 16;171(5):573–88. doi: 10.1002/ajmg.b.32346 (PMC4715802; doi:10.1002/ajmg.b.32346)
Supplement: Supplementary file 1 — Table SI. Lists of genes included in each of the gene‐set analyses. Table SII. Bivariate correlations of ODD subtypes. Table SIII. Top SNPs with p < 1.00E‐05 for association in the multivariate GWAS and their performance in univariate analysis. Table SIV. Index SNPs showing association with ODD at p < 1.00E‐04 after clumping of multivariate association results. Table SV. Top‐ranked genes previously implicated in the etiology of neurodevelopmental or neuropsychiatric disorders. Table SVI. SNPs showing association signal P<1.00E‐5 in the GWAS by Anney et al. (Anney et al., 2008) and their performance in our multivariate GWAS. Table SVII. List of ADHD GWAS top hits (p‐value < 1.00E‐05) that were compared with top hits from our study. Table SVIII. Genes/proteins/molecules from the molecular landscape linked to aggressive behavior through genetic and/or functional evidence. Figure S1. Outcome of the association analysis of the two ODD subtypes and the two ODD dimensions for all SNPs located in the OXTR region. Figure S2. Quantile quantile plot for the multivariate GWAS. Figure S3. Comparison with results of previous aggression related GWAS. Figure S4. Comparison with results of previous aggression related GWAS. [file AJMG-171-573-s001.doc]

**Supplement**

**Supplementary Table S1:** Lists of genes included in each of the gene-set analyses.

| **Dopamine gene-set** | | | | | **Serotonin gene-set** | | **Oxytocin gene-set** |
| --- | --- | --- | --- | --- | --- | --- | --- |
| ADCY1 | DRD3 | PPP1R14A | PPP2R2C | PRKAR2A | DDC | HTR6 | EZH2 |
| ADCY10 | DRD4 | PPP1R14B | PPP2R3A | PRKAR2B | GCH1 | HTR7 | GNAQ |
| ADCY2 | DRD5 | PPP1R14C | PPP2R4 | PTS | HTR1A | IL4I1 | GRK5 |
| ADCY3 | GCH1 | PPP1R14D | PPP2R5A | QDPR | HTR1B | PCBD1 | HTT |
| ADCY4 | IL4I1 | PPP1R1B | PPP2R5B | SLC18A1 | HTR1D | PTS | IGF1 |
| ADCY5 | NCS1 | PPP1R3A | PPP2R5C | SLC18A2 | HTR1E | QDPR | OXT |
| ADCY6 | PCBD1 | PPP1R3C | PPP2R5D | SLC18A3 | HTR2A | SLC18A1 | OXTR |
| ADCY7 | PPM1J | PPP1R3D | PPP2R5E | SLC6A3 | HTR2B | SLC18A2 | PPARA |
| ADCY8 | PPM1L | PPP1R7 | PRKACA | SMOX | HTR3A | SLC18A3 |  |
| ADCY9 | PPP1CA | PPP2CA | PRKACB | SPR | HTR3B | SLC6A4 |  |
| CALY/DRD1IP | PPP1CB | PPP2CB | PRKACG | TH | HTR3C | SMOX |  |
| COMT | PPP1CC | PPP2R1A | PRKAG1 |  | HTR3D | SPR |  |
| DDC | PPP1R10 | PPP2R1B | PRKAG2 |  | HTR3E | TPH1 |  |
| DRD1 | PPP1R11 | PPP2R2A | PRKAR1A |  | HTR4 | TPH2 |  |
| DRD2 | PPP1R12A | PPP2R2B | PRKAR1B |  | HTR5A |  |  |

**Supplementary Table S2**: Bivariate correlations of ODD subtypes.

|  | P1 | P2 | P3 | P4 | CTRS OPP | SDQ  CP | PACS ODD | PACS  CD |
| --- | --- | --- | --- | --- | --- | --- | --- | --- |
| P1 defiant vindictive dimension | 1 | 0.68 | 0.62 | 0.73 | 0.21 | 0.63 | 0.42 | 0.31 |
| P2 irritable dimension |  | 1 | 0.75 | 0.53 | 0.10 | 0.56 | 0.42 | 0.25 |
| P3 irritable/severe oppositionality |  | - | 1 | 0.57 | 0.14 | 0.52 | 0.38 | 0.23 |
| P4 severe oppositionality |  |  |  | 1 | 0.16 | 0.45 | 0.32 | 0.26 |

Note: CTRS OPP = Conners’ Teacher Rating Scale Oppositionality, SDQ CP = Strengths and Difficulties Questionnaire Conduct Problems, PACS = Parental Account of Childhood Symptoms, ODD = Oppositional Defiant Disorder, CD = Conduct Disorder, all correlations were significant at a threshold of p<0.05.

**Supplementary Table S3:** Top SNPs with *p* < 1.00E-05 for association in the multivariate GWAS and their performance in univariate analysis. Associated genes are presented for the SNPs that are located within exonic, intronic or untranslated regions of genes and for the SNPs located within 100 kilobases (kb) of downstream and upstream regions flanking a gene. The SNPs that do not fulfill these criteria are designated as 'intergenic'. SNPs with two genes located nearby are listed twice.

|  |  |  |  | **Multivariate analysis** | | | | |  |  | **Univariate analyses** | | | | | | | |
| --- | --- | --- | --- | --- | --- | --- | --- | --- | --- | --- | --- | --- | --- | --- | --- | --- | --- | --- |
|  |  |  |  |  | **P1** | **P2** | **P3** | **P4** |  |  | **P1** | | **P2** | | **P3** | | **P4** | |
| **CHR** | **SNP** | **BP *** | **Band** | **P value** | **loading** | **loading** | **loading** | **loading** | **Gene** | **Position ~ gene** | **Beta** | **P** | **Beta** | **P** | **OR** | **P** | **OR** | **P** |
| 3 | rs9822411 | 25338947 | p24.2 | 5.29E-06 | -0.04124 | -0.5858 | -0.7042 | 0.1419 | *RARB* | intronic | 0.07 | 8.25E-01 | 0.24 | 1.43E-03 | 1.95 | 1.79E-04 | 0.86 | 4.36E-01 |
| 3 | rs9833653 | 25344352 | p24.2 | 5.29E-06 | -0.04124 | -0.5858 | -0.7042 | 0.1419 | *RARB* | intronic | 0.07 | 8.25E-01 | 0.24 | 1.43E-03 | 1.95 | 1.79E-04 | 0.86 | 4.36E-01 |
| 8 | rs4500123 | 93039329 | q21.3 | 5.93E-06 | 0.5288 | 0.2728 | 0.3982 | 0.9613 | *RUNX1T1* | 3'UTR | 1.12 | 4.23E-03 | 0.13 | 1.41E-01 | 1.56 | 3.16E-02 | 2.82 | 4.39E-07 |
| 8 | rs4734962 | 93039798 | q21.3 | 5.93E-06 | 0.5288 | 0.2728 | 0.3982 | 0.9613 | *RUNX1T1* | 3'UTR | 1.12 | 4.23E-03 | 0.13 | 1.41E-01 | 1.56 | 3.16E-02 | 2.82 | 4.39E-07 |
| 9 | rs10821428 | 92227628 | q22.2 | 9.70E-06 | 0.3505 | 0.7513 | 0.2141 | 0.4764 | *(-)* | intergenic | 0.41 | 6.19E-02 | 0.20 | 6.48E-05 | 1.14 | 2.52E-01 | 1.36 | 1.08E-02 |
| 10 | rs1278352 | 127763366 | q26.2 | 1.24E-06 | -0.3312 | -0.8576 | -0.8092 | -0.1843 | *ADAM12* | intronic | 0.40 | 6.08E-02 | 0.24 | 9.40E-07 | 1.67 | 4.97E-06 | 1.13 | 2.88E-01 |
| 11 | rs4758132 | 6739977 | p15.4 | 3.15E-06 | 0.002295 | 0.3843 | 0.7613 | 0.3524 | *OR2AG2* | 5 kb downstream | 0.00 | 9.90E-01 | -0.18 | 3.38E-02 | 0.44 | 3.33E-05 | 0.65 | 5.24E-02 |
| 11 | rs4758132 | 6739977 | p15.4 | 3.15E-06 | 0.002295 | 0.3843 | 0.7613 | 0.3524 | *OR2AG1* | 23 kb upstream | 0.00 | 9.90E-01 | -0.18 | 3.38E-02 | 0.44 | 3.33E-05 | 0.65 | 5.24E-02 |
| 12 | rs17399946 | 23968776 | p12.1 | 6.95E-06 | 0.2993 | 0.6779 | 0.5185 | 0.7186 | *SOX5* | intronic | -0.49 | 1.05E-01 | -0.25 | 2.58E-04 | 0.65 | 5.16E-03 | 0.49 | 1.48E-04 |
| 12 | rs10784227 | 60178960 | q14.1 | 5.77E-06 | 0.07303 | 0.06676 | 0.6157 | -0.1548 | *(-)* | intergenic | 0.09 | 6.96E-01 | 0.02 | 7.38E-01 | 1.52 | 8.68E-04 | 0.89 | 3.99E-01 |
| 12 | rs1453714 | 60187685 | q14.1 | 4.74E-06 | 0.05256 | 0.06777 | 0.6182 | -0.162 | *(-)* | intergenic | 0.06 | 8.11E-01 | 0.02 | 7.64E-01 | 1.53 | 7.69E-04 | 0.88 | 3.46E-01 |
| 12 | rs12370275 | 60193289 | q14.1 | 2.41E-06 | 0.0263 | 0.03506 | 0.6045 | -0.1592 | *(-)* | intergenic | 0.01 | 9.51E-01 | 0.01 | 9.07E-01 | 1.53 | 7.45E-04 | 0.88 | 3.35E-01 |
| 12 | rs11174055 | 60195641 | q14.1 | 2.41E-06 | 0.0263 | 0.03506 | 0.6045 | -0.1592 | *(-)* | intergenic | 0.01 | 9.51E-01 | 0.01 | 9.07E-01 | 1.53 | 7.45E-04 | 0.88 | 3.35E-01 |
| 12 | rs1453708 | 60200018 | q14.1 | 2.49E-06 | -0.0328 | -0.03922 | -0.6107 | 0.1501 | *(-)* | intergenic | 0.02 | 9.20E-01 | 0.01 | 8.88E-01 | 1.54 | 6.62E-04 | 0.88 | 3.64E-01 |
| 14 | rs11844114 | 93530282 | q32.13 | 4.52E-06 | -0.1947 | -0.3355 | -0.5466 | 0.3344 | *DDX24* | 54 kb downstream | 0.23 | 2.92E-01 | 0.09 | 6.84E-02 | 1.39 | 3.00E-03 | 0.80 | 6.69E-02 |
| 14 | rs11844114 | 93530282 | q32.13 | 4.52E-06 | -0.1947 | -0.3355 | -0.5466 | 0.3344 | *ASB2* | 17 kb upstream | 0.23 | 2.92E-01 | 0.09 | 6.84E-02 | 1.39 | 3.00E-03 | 0.80 | 6.69E-02 |
| 14 | rs4905137 | 93535834 | q32.13 | 7.49E-06 | -0.01004 | -0.1563 | -0.5016 | 0.4062 | *DDX24* | 49 kb downstream | 0.01 | 9.58E-01 | 0.04 | 4.05E-01 | 1.34 | 7.49E-03 | 0.77 | 2.92E-02 |
| 14 | rs4905137 | 93535834 | q32.13 | 7.49E-06 | -0.01004 | -0.1563 | -0.5016 | 0.4062 | *ASB2* | 23 kb upstream | 0.01 | 9.58E-01 | 0.04 | 4.05E-01 | 1.34 | 7.49E-03 | 0.77 | 2.92E-02 |
| 16 | rs7204436 | 5223492 | p13.3 | 1.98E-07 | -0.1103 | 0.2759 | 0.2562 | 0.5517 | *(-)* | intergenic | -0.15 | 5.23E-01 | 0.09 | 8.95E-02 | 1.20 | 1.14E-01 | 1.53 | 8.25E-04 |
| 20 | rs6088857 | 29690425 | q11.21 | 9.88E-06 | 0.7255 | 0.5855 | 0.5625 | 0.9935 | *COX4I2* | intronic | 0.98 | 1.13E-04 | 0.18 | 1.87E-03 | 1.49 | 2.92E-03 | 2.04 | 2.20E-07 |
| 20 | rs6088864 | 29691818 | q11.21 | 9.88E-06 | 0.7255 | 0.5855 | 0.5625 | 0.9935 | *COX4I2* | intronic | 0.98 | 1.13E-04 | 0.18 | 1.87E-03 | 1.49 | 2.92E-03 | 2.04 | 2.20E-07 |
| 20 | rs6060446 | 29695298 | q11.21 | 9.88E-06 | 0.7255 | 0.5855 | 0.5625 | 0.9935 | *COX4I2* | intronic | 0.98 | 1.13E-04 | 0.18 | 1.87E-03 | 1.49 | 2.92E-03 | 2.04 | 2.20E-07 |
| 20 | rs6120970 | 29696113 | q11.21 | 6.42E-06 | 0.7426 | 0.5865 | 0.5787 | 0.995 | *COX4I2* | intronic | 1.02 | 6.03E-05 | 0.18 | 1.56E-03 | 1.52 | 1.91E-03 | 2.07 | 1.42E-07 |
| 20 | rs7272062 | 29722806 | q11.21 | 4.44E-06 | 0.6186 | 0.4683 | 0.4755 | 0.989 | *BCL2L1* | intronic | 0.85 | 6.63E-04 | 0.14 | 1.08E-02 | 1.40 | 9.07E-03 | 2.08 | 8.70E-08 |
| 20 | rs6119651 | 29722907 | q11.21 | 4.44E-06 | 0.6186 | 0.4683 | 0.4755 | 0.989 | *BCL2L1* | intronic | 0.85 | 6.63E-04 | 0.14 | 1.08E-02 | 1.40 | 9.07E-03 | 2.08 | 8.70E-08 |
| 20 | rs6060763 | 29745884 | q11.21 | 3.82E-06 | 0.608 | 0.4439 | 0.4689 | 0.9882 | *BCL2L1* | intronic | 0.84 | 7.58E-04 | 0.14 | 1.41E-02 | 1.40 | 9.55E-03 | 2.08 | 7.77E-08 |
| 20 | rs1994251 | 29750989 | q11.21 | 3.82E-06 | 0.608 | 0.4439 | 0.4689 | 0.9882 | *BCL2L1* | intronic | 0.84 | 7.58E-04 | 0.14 | 1.41E-02 | 1.40 | 9.55E-03 | 2.08 | 7.77E-08 |
| 20 | rs1994250 | 29751155 | q11.21 | 3.82E-06 | 0.608 | 0.4439 | 0.4689 | 0.9882 | *BCL2L1* | intronic | 0.84 | 7.58E-04 | 0.14 | 1.41E-02 | 1.40 | 9.55E-03 | 2.08 | 7.77E-08 |
| 20 | rs6060793 | 29752491 | q11.21 | 3.82E-06 | 0.608 | 0.4439 | 0.4689 | 0.9882 | *BCL2L1* | intronic | 0.84 | 7.58E-04 | 0.14 | 1.41E-02 | 1.40 | 9.55E-03 | 2.08 | 7.77E-08 |
| 20 | rs6060812 | 29756464 | q11.21 | 3.82E-06 | 0.608 | 0.4439 | 0.4689 | 0.9882 | *BCL2L1* | intronic | 0.84 | 7.58E-04 | 0.14 | 1.41E-02 | 1.40 | 9.55E-03 | 2.08 | 7.77E-08 |
| 20 | rs6060821 | 29757695 | q11.21 | 3.82E-06 | 0.608 | 0.4439 | 0.4689 | 0.9882 | *BCL2L1* | intronic | 0.84 | 7.58E-04 | 0.14 | 1.41E-02 | 1.40 | 9.55E-03 | 2.08 | 7.77E-08 |
| 20 | rs6058421 | 29758674 | q11.21 | 3.82E-06 | 0.608 | 0.4439 | 0.4689 | 0.9882 | *BCL2L1* | intronic | 0.84 | 7.58E-04 | 0.14 | 1.41E-02 | 1.40 | 9.55E-03 | 2.08 | 7.77E-08 |
| 20 | rs7354225 | 29760542 | q11.21 | 3.82E-06 | 0.608 | 0.4439 | 0.4689 | 0.9882 | *BCL2L1* | intronic | 0.84 | 7.58E-04 | 0.14 | 1.41E-02 | 1.40 | 9.55E-03 | 2.08 | 7.77E-08 |
| 20 | rs6060870 | 29766960 | q11.21 | 3.82E-06 | 0.608 | 0.4439 | 0.4689 | 0.9882 | *BCL2L1* | intronic | 0.84 | 7.58E-04 | 0.14 | 1.41E-02 | 1.40 | 9.55E-03 | 2.08 | 7.77E-08 |
| 20 | rs3181073 | 29770860 | q11.21 | 3.82E-06 | 0.608 | 0.4439 | 0.4689 | 0.9882 | *BCL2L1* | intronic | 0.84 | 7.58E-04 | 0.14 | 1.41E-02 | 1.40 | 9.55E-03 | 2.08 | 7.77E-08 |
| 20 | rs2376996 | 29780328 | q11.21 | 3.63E-06 | 0.612 | 0.4547 | 0.4644 | 0.9876 | *COX4I2* | 84 kb downstream | 0.85 | 7.05E-04 | 0.14 | 1.22E-02 | 1.40 | 1.04E-02 | 2.08 | 8.33E-08 |
| 20 | rs2376996 | 29780328 | q11.21 | 3.63E-06 | 0.612 | 0.4547 | 0.4644 | 0.9876 | *BCL2L1* | 6 kb upstream | 0.85 | 7.05E-04 | 0.14 | 1.22E-02 | 1.40 | 1.04E-02 | 2.08 | 8.33E-08 |
| 20 | rs6089055 | 29787706 | q11.21 | 3.63E-06 | 0.612 | 0.4547 | 0.4644 | 0.9876 | *COX4I2* | 91 kb downstream | 0.85 | 7.05E-04 | 0.14 | 1.22E-02 | 1.40 | 1.04E-02 | 2.08 | 8.33E-08 |
| 20 | rs6089055 | 29787706 | q11.21 | 3.63E-06 | 0.612 | 0.4547 | 0.4644 | 0.9876 | *TPX2* | 3 kb upstream | 0.85 | 7.05E-04 | 0.14 | 1.22E-02 | 1.40 | 1.04E-02 | 2.08 | 8.33E-08 |
| 20 | rs6060912 | 29789416 | q11.21 | 6.61E-06 | 0.6199 | 0.4657 | 0.4838 | 0.9901 | *COX4I2* | 93 kb downstream | 0.84 | 7.95E-04 | 0.14 | 1.22E-02 | 1.40 | 9.02E-03 | 2.05 | 1.39E-07 |
| 20 | rs6060912 | 29789416 | q11.21 | 6.61E-06 | 0.6199 | 0.4657 | 0.4838 | 0.9901 | *TPX2* | 1 kb upstream | 0.84 | 7.95E-04 | 0.14 | 1.22E-02 | 1.40 | 9.02E-03 | 2.05 | 1.39E-07 |
| 20 | rs6089058 | 29789999 | q11.21 | 6.87E-06 | 0.6134 | 0.4555 | 0.4796 | 0.9893 | *COX4I2* | 94 kb downstream | 0.83 | 8.85E-04 | 0.14 | 1.38E-02 | 1.40 | 9.53E-03 | 2.05 | 1.44E-07 |
| 20 | rs6089058 | 29789999 | q11.21 | 6.87E-06 | 0.6134 | 0.4555 | 0.4796 | 0.9893 | *TPX2* | 566 bp upstream | 0.83 | 8.85E-04 | 0.14 | 1.38E-02 | 1.40 | 9.53E-03 | 2.05 | 1.44E-07 |
| 20 | rs6058448 | 29796057 | q11.21 | 9.87E-06 | 0.6208 | 0.4391 | 0.4712 | 0.991 | *TPX2* | intronic | 0.84 | 8.50E-04 | 0.14 | 1.85E-02 | 1.39 | 1.17E-02 | 2.05 | 1.93E-07 |
| 20 | rs6060923 | 29800316 | q11.21 | 6.87E-06 | 0.6134 | 0.4555 | 0.4796 | 0.9893 | *TPX2* | intronic | 0.83 | 8.85E-04 | 0.14 | 1.38E-02 | 1.40 | 9.53E-03 | 2.05 | 1.44E-07 |
| 20 | rs6058450 | 29807572 | q11.21 | 6.87E-06 | 0.6134 | 0.4555 | 0.4796 | 0.9893 | *TPX2* | intronic | 0.83 | 8.85E-04 | 0.14 | 1.38E-02 | 1.40 | 9.53E-03 | 2.05 | 1.44E-07 |
| 20 | rs6089070 | 29841434 | q11.21 | 6.87E-06 | 0.6134 | 0.4555 | 0.4796 | 0.9893 | *TPX2* | intronic | 0.83 | 8.85E-04 | 0.14 | 1.38E-02 | 1.40 | 9.53E-03 | 2.05 | 1.44E-07 |
| 20 | rs6089071 | 29842045 | q11.21 | 6.87E-06 | 0.6134 | 0.4555 | 0.4796 | 0.9893 | *TPX2* | intronic | 0.83 | 8.85E-04 | 0.14 | 1.38E-02 | 1.40 | 9.53E-03 | 2.05 | 1.44E-07 |
| 20 | rs3203770 | 29845976 | q11.21 | 4.86E-06 | 0.6053 | 0.4463 | 0.4686 | 0.9871 | *TPX2* | synonymous coding | 0.83 | 8.82E-04 | 0.14 | 1.49E-02 | 1.39 | 1.06E-02 | 2.06 | 1.21E-07 |
| 20 | rs6058463 | 29848853 | q11.21 | 4.08E-06 | 0.6039 | 0.4477 | 0.474 | 0.9871 | *TPX2* | intronic | 0.83 | 8.47E-04 | 0.14 | 1.42E-02 | 1.40 | 9.73E-03 | 2.07 | 1.03E-07 |
| 20 | rs6119725 | 29849218 | q11.21 | 4.08E-06 | 0.6039 | 0.4477 | 0.474 | 0.9871 | *TPX2* | intronic | 0.83 | 8.47E-04 | 0.14 | 1.42E-02 | 1.40 | 9.73E-03 | 2.07 | 1.03E-07 |
| 20 | rs6060960 | 29858178 | q11.21 | 3.00E-06 | 0.6045 | 0.4489 | 0.4816 | 0.9877 | *TPX2* | 5 kb downstream | 0.84 | 7.68E-04 | 0.14 | 1.29E-02 | 1.41 | 8.07E-03 | 2.08 | 8.08E-08 |
| 20 | rs6060960 | 29858178 | q11.21 | 3.00E-06 | 0.6045 | 0.4489 | 0.4816 | 0.9877 | *MYLK2* | 13 kb upstream | 0.84 | 7.68E-04 | 0.14 | 1.29E-02 | 1.41 | 8.07E-03 | 2.08 | 8.08E-08 |
| 20 | rs6121242 | 29874538 | q11.21 | 9.79E-06 | 0.626 | 0.4589 | 0.4747 | 0.9905 | *MYLK2* | intronic | 0.84 | 8.41E-04 | 0.14 | 1.51E-02 | 1.38 | 1.25E-02 | 2.03 | 2.40E-07 |
| 20 | rs6119729 | 29876824 | q11.21 | 9.79E-06 | 0.626 | 0.4589 | 0.4747 | 0.9905 | *MYLK2* | intronic | 0.84 | 8.41E-04 | 0.14 | 1.51E-02 | 1.38 | 1.25E-02 | 2.03 | 2.40E-07 |
| 20 | rs6060979 | 29881183 | q11.21 | 8.07E-06 | 0.624 | 0.4533 | 0.4737 | 0.9904 | *MYLK2* | intronic | 0.85 | 7.98E-04 | 0.14 | 1.58E-02 | 1.39 | 1.20E-02 | 2.05 | 1.90E-07 |
| 20 | rs4518038 | 29882712 | q11.21 | 8.07E-06 | 0.624 | 0.4533 | 0.4737 | 0.9904 | *MYLK2* | intronic | 0.85 | 7.98E-04 | 0.14 | 1.58E-02 | 1.39 | 1.20E-02 | 2.05 | 1.90E-07 |
| 20 | rs17093657 | 29892555 | q11.21 | 8.07E-06 | 0.624 | 0.4533 | 0.4737 | 0.9904 | *FOXS1* | 3 kb downstream | 0.85 | 7.98E-04 | 0.14 | 1.58E-02 | 1.39 | 1.20E-02 | 2.05 | 1.90E-07 |
| 20 | rs17093657 | 29892555 | q11.21 | 8.07E-06 | 0.624 | 0.4533 | 0.4737 | 0.9904 | *TTLL9* | 30 kb upstream | 0.85 | 7.98E-04 | 0.14 | 1.58E-02 | 1.39 | 1.20E-02 | 2.05 | 1.90E-07 |
| 20 | rs6060989 | 29893424 | q11.21 | 8.07E-06 | 0.624 | 0.4533 | 0.4737 | 0.9904 | *FOXS1* | 2 kb downstream | 0.85 | 7.98E-04 | 0.14 | 1.58E-02 | 1.39 | 1.20E-02 | 2.05 | 1.90E-07 |
| 20 | rs6060989 | 29893424 | q11.21 | 8.07E-06 | 0.624 | 0.4533 | 0.4737 | 0.9904 | *TTLL9* | 29 kb upstream | 0.85 | 7.98E-04 | 0.14 | 1.58E-02 | 1.39 | 1.20E-02 | 2.05 | 1.90E-07 |
| 20 | rs6060992 | 29894916 | q11.21 | 6.26E-06 | 0.6228 | 0.4548 | 0.4758 | 0.9902 | *FOXS1* | 848 bp downstream | 0.85 | 7.26E-04 | 0.14 | 1.45E-02 | 1.39 | 1.08E-02 | 2.06 | 1.44E-07 |
| 20 | rs6060992 | 29894916 | q11.21 | 6.26E-06 | 0.6228 | 0.4548 | 0.4758 | 0.9902 | *TTLL9* | 27 kb upstream | 0.85 | 7.26E-04 | 0.14 | 1.45E-02 | 1.39 | 1.08E-02 | 2.06 | 1.44E-07 |
| 20 | rs6089094 | 29895432 | q11.21 | 6.26E-06 | 0.6228 | 0.4548 | 0.4758 | 0.9902 | *FOXS1* | 332 bp downstream | 0.85 | 7.26E-04 | 0.14 | 1.45E-02 | 1.39 | 1.08E-02 | 2.06 | 1.44E-07 |
| 20 | rs6089094 | 29895432 | q11.21 | 6.26E-06 | 0.6228 | 0.4548 | 0.4758 | 0.9902 | *TTLL9* | 27 kb upstream | 0.85 | 7.26E-04 | 0.14 | 1.45E-02 | 1.39 | 1.08E-02 | 2.06 | 1.44E-07 |

Note: P1= defiant vindictive dimension, P2= irritable dimension, P3= irritable/severe oppositionality, P4= severe oppositionality. * Positions according to HAPMAP 2 Release 22.

**Supplementary Table S4:** Index SNPs showing association with ODD at *p* < 1.00E-04 after clumping of multivariate association results. Only SNPs that are located within exonic, intronic or untranslated regions of genes, or that are located within 100 kilobases (kb) of downstream and upstream regions flanking a gene are shown. The genes in bold have already been implicated in the etiology of neurodevelopmental or neuropsychiatric disorders.

| **CHR** | **SNP** | **BP *** | **Band** | **P value** | **N clumped**** | **Gene** | **Position ~ gene** |  | **Gene name** |
| --- | --- | --- | --- | --- | --- | --- | --- | --- | --- |
| 1 | rs11806028 | 81768458 | p31.1 | 3.57E-05 | 17 | LPHN2 | intronic |  | latrophilin 2 |
| 1 | rs10910623 | 231005322 | q42.2 | 3.88E-05 | 38 | MAP10 | 2 kb upstream |  | microtubule-associated protein 10 |
| 1 | rs7542425 | 245616954 | q44 | 8.49E-05 | 5 | **NLRP3** | 29 kb upstream |  | NLR family, pyrin domain containing 3 |
| 1 | rs7542425 | 245616954 | q44 | 8.49E-05 | 5 | OR2B11 | 64 kb downstream |  | olfactory receptor, family 2, subfamily B, member 11 |
| 2 | rs17034837 | 68089376 | p14 | 7.00E-05 | 1 | C1D | 32 kb downstream |  | C1D nuclear receptor corepressor |
| 2 | rs6712239 | 168557693 | q24.3 | 1.07E-05 | 65 | **STK39** | intronic |  | serine threonine kinase 39 |
| 2 | rs6710235 | 168622325 | q24.3 | 9.66E-05 | 2 | **STK39** | intronic |  | serine threonine kinase 39 |
| 3 | rs9822411 | 25338947 | p24.2 | 5.29E-06 | 41 | **RARB** | intronic |  | retinoic acid receptor, beta |
| 4 | rs11732351 | 8006388 | p16.1 | 9.08E-05 | 41 | ABLIM2 | 12 kb downstream |  | actin binding LIM protein family, member 2 |
| 4 | rs11732351 | 8006388 | p16.1 | 9.08E-05 | 41 | AFAP1 | 14 kb upstream |  | actin filament associated protein 1 |
| 4 | rs11724348 | 25593163 | p15.2 | 7.72E-05 | 1 | SMIM20 | 53 kb downstream |  | small integral membrane protein 20 |
| 5 | rs13188386 | 42509312 | p13.1 | 7.06E-05 | 200 | GHR | intronic |  | growth hormone receptor |
| 5 | rs3797207 | 95195665 | q15 | 5.12E-05 | 18 | **GLRX** | 11 kb upstream |  | glutaredoxin (thioltransferase) |
| 5 | rs3797207 | 95195665 | q15 | 5.12E-05 | 18 | **RHOBTB3** | 10 kb downstream |  | Rho-related BTB domain containing 3 |
| 5 | rs2973725 | 177736638 | q35.3 | 6.14E-05 | 23 | COL23A1 | intronic |  | collagen, type XXIII, alpha 1 |
| 6 | rs11967472 | 31379264 | p21.33 | 7.55E-05 | 110 | **HLA-C** | intronic |  | major histocompatibility complex, class I, C |
| 6 | rs7739455 | 74477349 | q13 | 9.83E-05 | 82 | CD109 | intronic |  | CD109 molecule |
| 7 | rs7812175 | 16447568 | p21.2 | 6.81E-05 | 52 | ISPD | 20 kb upstream |  | isoprenoid synthase domain containing |
| 7 | rs7812175 | 16447568 | p21.1 | 6.81E-05 | 52 | SOSTDC1 | 20 kb downstream |  | sclerostin domain containing 1 |
| 7 | rs7811079 | 31909609 | p14.3 | 1.13E-05 | 37 | PDE1C | intronic |  | phosphodiesterase 1C, calmodulin-dependent 70kDa |
| 7 | rs1721393 | 38043347 | p14.1 | 6.95E-05 | 68 | EPDR1 | 85 kb downstream |  | ependymin related 1 |
| 7 | rs1721393 | 38043347 | p14.1 | 6.95E-05 | 68 | SFRP4 | 12 kb upstream |  | secreted frizzled-related protein 4 |
| 8 | rs2013938 | 6463312 | p23.1 | 1.31E-05 | 29 | **MCPH1** | intronic |  | microcephalin 1 |
| 8 | rs4500123 | 93039329 | q21.3 | 5.93E-06 | 3 | RUNX1T1 | 3'UTR |  | runt-related transcription factor 1; translocated to, 1 (cyclin D-related) |
| 9 | rs4075163 | 90788923 | q22.1 | 5.48E-05 | 14 | C9orf47 | 7 kb upstream |  | chromosome 9 open reading frame 47 |
| 9 | rs4075163 | 90788923 | q22.1 | 5.48E-05 | 14 | **SHC3** | 29 kb downstream |  | SHC (Src homology 2 domain containing) transforming protein 3 |
| 9 | rs2479828 | 98090615 | q22.32 | 5.59E-05 | 0 | HSD17B3 | intronic |  | hydroxysteroid (17-beta) dehydrogenase 3 |
| 9 | rs13291423 | 132860593 | q34.12 | 7.55E-05 | 2 | **LAMC3** | 14 kb upstream |  | laminin, gamma 3 |
| 10 | rs4919079 | 98927898 | q24.1 | 7.93E-05 | 15 | SLIT1 | intronic |  | slit homolog 1 (Drosophila) |
| 10 | rs1278352 | 127763366 | q26.2 | 1.24E-06 | 42 | **ADAM12** | intronic |  | ADAM metallopeptidase domain 12 |
| 11 | rs4758132 | 6739977 | p15.4 | 3.15E-06 | 23 | OR2AG1 | 23 kb upstream |  | olfactory receptor, family 2, subfamily AG, member 1 |
| 11 | rs4758132 | 6739977 | p15.4 | 3.15E-06 | 23 | OR2AG2 | 6 kb downstream |  | olfactory receptor, family 2, subfamily AG, member 2 |
| 11 | rs4945343 | 78732638 | q14.1 | 6.67E-05 | 6 | TENM4 | intronic |  | teneurin transmembrane protein 4 |
| 11 | rs10895301 | 101869559 | q22.2 | 7.29E-05 | 30 | MMP7 | 27 kb downstream |  | matrix metallopeptidase 7 (matrilysin, uterine) |
| 11 | rs10895301 | 101869559 | q22.2 | 7.29E-05 | 30 | TMEM123 | 41 kb upstream |  | transmembrane protein 123 |
| 11 | rs17116334 | 113466972 | q23.2 | 2.77E-05 | 8 | ZBTB16 | intronic |  | zinc finger and BTB domain containing 16 |
| 12 | rs2159381 | 3222193 | p13.33 | 6.06E-05 | 5 | TSPAN9 | intronic |  | tetraspanin 9 |
| 12 | rs17399946 | 23968776 | p12.1 | 6.95E-06 | 14 | **SOX5** | intronic |  | SRY (sex determining region Y)-box 5 |
| 13 | rs1005720 | 28981187 | q12.3 | 8.30E-05 | 46 | SLC7A1 | 360 bp downstream |  | solute carrier family 7 (cationic amino acid transporter, y+ system), member 1 |
| 13 | rs10507466 | 37361657 | q13.3 | 8.68E-05 | 82 | TRPC4 | 20 kb upstream |  | transient receptor potential cation channel, subfamily C, member 4 |
| 13 | rs1330598 | 60831132 | q21.2 | 2.39E-05 | 21 | PCDH20 | 51 kb downstream |  | protocadherin 20 |
| 14 | rs11844114 | 93530282 | q32.12 | 4.52E-06 | 11 | ASB2 | 17 kb upstream |  | ankyrin repeat and SOCS box containing 2 |
| 14 | rs11844114 | 93530282 | q32.12 | 4.52E-06 | 11 | DDX24 | 54 kb downstream |  | DEAD (Asp-Glu-Ala-Asp) box helicase 24 |
| 15 | rs4275799 | 56693437 | q21.3 | 6.18E-05 | 132 | **ADAM10** | intronic |  | ADAM metallopeptidase domain 10 |
| 15 | rs7164017 | 60675326 | q22.2 | 3.57E-05 | 2 | TLN2 | intronic |  | talin 2 |
| 15 | rs950168 | 82497201 | q25.2 | 4.07E-05 | 10 | **ADAMTSL3** | intronic |  | ADAMTS-like 3 |
| 16 | rs1862861 | 4195458 | p13.3 | 7.88E-05 | 6 | SRL | intronic |  | sarcalumenin |
| 17 | rs17837003 | 29264945 | q12 | 6.51E-05 | 13 | **ASIC2** | intronic |  | acid-sensing (proton-gated) ion channel 2 |
| 17 | rs590040 | 74030083 | q25.3 | 4.02E-05 | 10 | DNAH17 | intronic |  | dynein, axonemal, heavy chain 17 |
| 20 | rs6108320 | 9581004 | p12.2 | 6.24E-05 | 34 | **PAK7** | intronic |  | p21 protein (Cdc42/Rac)-activated kinase 7 |
| 20 | rs6060960 | 29858178 | q11.21 | 3.00E-06 | 150 | MYLK2 | 13 kb upstream |  | myosin light chain kinase 2 |
| 20 | rs6060960 | 29858178 | q11.21 | 3.00E-06 | 150 | TPX2 | 5 kb downstream |  | TPX2, microtubule-associated |
| 20 | rs6061345 | 59762911 | q13.33 | 4.04E-05 | 16 | CDH4 | intronic |  | cadherin 4, type 1, R-cadherin |
| 21 | rs2839417 | 42192806 | q22.3 | 7.00E-05 | 12 | C2CD2 | intronic |  | C2 calcium-dependent domain containing 2 |

Note: * Positions according to HAPMAP 2 Release 22. ** Total number of SNPs in clump passing LD (r^2^=.2) and physical distance (10,000 kb) thresholds for clumping.

**Supplementary Table S5:** Top-ranked genes previously implicated in the etiology of neurodevelopmental or neuropsychiatric disorders.

| **Gene** | **Locus** | **Associated with neurodevelopmental or neuropsychiatric disorders** |
| --- | --- | --- |
| *ADAM10* | 15q21.3 | *ADAM10* mutations were found in 11 out of 16 affected individuals from families with late-onset Alzheimer's disease (AD) . |
| *ADAM12* | 10q26.2 | *ADAM12* resides in a genetic locus that has been linked to schizophrenia (SZ) and reduced numerical density of ADAM12-expressing oligodendrocytes was found in the white matter of SZ patients . Significant interaction was found between rs3740473 (in *SH3PXD2A*) and rs11244787 (in *ADAM12*) with respect to the risk of developing AD , but this interaction was not replicated in a subsequent study . ADAM12 levels in urine of bipolar disorder (BPD) patients are elevated compared to healthy controls . |
| *ADAMTSL3* | 15q25.2 | The *ADAMTSL3*-located SNPs rs2135551, rs950169 and rs1911155 yielded P-values just below genome-wide significance in a GWAS of SZ , with rs950169 as the most likely causative variant for the GWAS association . |
| *ASIC2* | 17q12 | SNPs in *ASIC2* showed association with panic disorder in a Faroese case-control sample, but not in an independent Danish sample . |
| *GLRX* | 5q15 | The Glrx protein expression pattern in hippocampus tissue was shown to be altered in AD patients compared to controls . |
| *HLA-C* | 6p21.33 | Various strong association signals for SNPs spanning the Major Histocompatibility Complex (MHC) region have been found in SZ GWASs and HLA-C*01:02 has been specifically associated with SZ . |
| *LAMC3* | 9q34.12 | Exome sequencing studies of parent-child trios exhibiting sporadic autism spectrum disorders (ASDs) identified de novo mutations in *LAMC3*, implying involvement of this gene in ASD etiology . |
| *MCPH1* | 8p23.1 | *MCPH1* has been implicated in Autosomal recessive primary microcephaly (MCPH) with mental retardation . |
| *NLRP3* | 1q44 | Activation of the NLRP3-dependent inflammasome was demonstrated in blood mononuclear cells from depressive patients . NLRP3 polymorphisms have been associated with late-onset AD in a candidate gene association study |
| *PAK7* | 20p12.2 | A duplication overlapping *PAK7* showed association with psychosis . |
| *RARB* | 3p24.2 | The levels of RARB were found to be lower in patients with major depression than in healthy controls . |
| *RHOBTB3* | 5q15 | A study using gene expression profiling to identify blood biomarkers for psychotic symptoms found decreased expression of *RHOBTB3* in patients with SZ or related disorders with high hallucinations states . |
| *SHC3* | 9q22.1 | SNP-based and haplotype analyses show associations of *SHC3* variants with nicotine dependence . |
| *SOX5* | 12p12.1 | Copy number variations in *SOX5* have been found in patients with ASDs . *SOX5* haploinsufficiency was also found in several patients presenting with with speech delay, intellectual disability and behavior abnormalities . |
| *STK39* | 2q24.3 | Analysis of candidate autism loci on chromosome 2q24-q33 showed that different haplotypes of SNPs encompassing *STK39* are significantly associated with autism . The STK39 locus shows genome-wide significant association with Parkinson's disease (PD) risk in large-scale meta-analyses of PD GWAS and risk gene studies . |

**Supplementary Table S6:** SNPs showing association signal P<1.00E-5 in the GWAS by Anney et al. (Anney et al., 2008) and their performance in our multivariate GWAS.

| CHR | SNP | BP | P value |
| --- | --- | --- | --- |
| 9 | rs10815798 | 8225633 | 1.51E-02 |
| 3 | rs13061352 | 22203581 | 1.69E-02 |
| 1 | rs2064648 | 30410603 | 2.43E-02 |
| 1 | rs2180233 | 30400299 | 2.89E-02 |
| 1 | rs4949546 | 30406270 | 2.89E-02 |
| 1 | rs1543424 | 30392051 | 3.31E-02 |
| 7 | rs10229603 | 112415609 | 5.07E-02 |
| 1 | rs6661210 | 155399012 | 5.73E-02 |
| 1 | rs10796972 | 155399761 | 5.73E-02 |
| 1 | rs6700498 | 155399774 | 5.73E-02 |
| 1 | rs1176542 | 155400100 | 5.73E-02 |
| 1 | rs1176543 | 155400219 | 5.73E-02 |
| 1 | rs1176551 | 155402280 | 5.73E-02 |
| 1 | rs1176555 | 155403165 | 5.73E-02 |
| 11 | rs1557488 | 126124400 | 5.87E-02 |
| 11 | rs1557487 | 126124791 | 5.87E-02 |
| 11 | rs10831284 | 94307612 | 6.04E-02 |
| 1 | rs11264625 | 155395329 | 6.14E-02 |
| 1 | rs6427356 | 155397190 | 6.14E-02 |
| 16 | rs4889240 | 79714023 | 8.80E-02 |
| 11 | rs10736554 | 126120771 | 9.29E-02 |
| 8 | rs4734494 | 101986897 | 9.56E-02 |
| 8 | rs4734495 | 101986993 | 9.62E-02 |
| 8 | rs931812 | 101988497 | 9.62E-02 |
| 1 | rs701157 | 228741449 | 1.48E-01 |
| 12 | rs789560 | 68618094 | 1.64E-01 |
| 14 | rs1951082 | 26329883 | 1.92E-01 |
| 14 | rs8021717 | 26333357 | 1.98E-01 |
| 10 | rs2764978 | 3273385 | 2.11E-01 |
| 10 | rs2764980 | 3274007 | 2.11E-01 |
| 10 | rs2814925 | 3274061 | 2.11E-01 |
| 2 | rs1521883 | 202657917 | 2.40E-01 |
| 1 | rs10797919 | 182119537 | 2.44E-01 |
| 1 | rs4079923 | 182113907 | 2.53E-01 |
| 15 | rs4533251 | 95063431 | 2.81E-01 |
| 16 | rs16973500 | 70522697 | 2.85E-01 |
| 12 | rs7297018 | 78586361 | 3.30E-01 |
| 2 | rs939745 | 202649555 | 3.66E-01 |
| 2 | rs1521882 | 202658096 | 3.67E-01 |
| 2 | rs1521879 | 202661472 | 3.72E-01 |
| 4 | rs6536350 | 159660267 | 3.79E-01 |
| 13 | rs10492664 | 107614226 | 4.32E-01 |
| 13 | rs8002852 | 107616886 | 4.70E-01 |
| 18 | rs7236632 | 53585200 | 5.07E-01 |
| 2 | rs6733379 | 34333579 | 5.51E-01 |
| 2 | rs7595103 | 77928752 | 6.93E-01 |
| 13 | rs9512900 | 27327738 | 7.16E-01 |
| 5 | rs1644305 | 133231495 | 7.40E-01 |
| 5 | rs1644308 | 133227448 | 7.55E-01 |
| 21 | rs2826340 | 20807173 | 8.38E-01 |
| 16 | rs1381102 | 62512948 | 8.54E-01 |
| 2 | rs1487044 | 77918912 | 9.54E-01 |
| 2 | rs1487045 | 77918966 | 9.54E-01 |
| 16 | rs12921846 | - | - |

**Supplementary Table S7:** List of ADHD GWAS top hits (p-value < 1.00E-05) that were compared with top hits from our study.

| **ADHD GWAS** | **SNP** | **p-value** |
| --- | --- | --- |
|  | rs5016282 | 1.00E-06 |
|  | rs2556378 | 7.00E-06 |
|  | rs7984422 | 5.08E-06 |
|  | rs10838881 | 9.29E-06 |
|  | rs2509689 | 5.62E-06 |
|  | rs8047014 | 4.00E-06 |
|  | rs2290416 | 9.00E-06 |
|  | rs4147141 | 6.00E-06 |
|  | rs1350666 | 8.00E-06 |
|  | rs11719664 | 2.00E-06 |
|  | rs130575 | 5.00E-06 |
|  | rs260461 | 8.00E-06 |
|  | rs7577925 | 3.00E-06 |
|  | rs930421 | 6.00E-06 |
|  | rs1514928 | 3.00E-06 |
|  | rs7992643 | 5.00E-06 |
|  | rs10767942 | 8.00E-06 |
|  | rs6791644 | 8.00E-06 |
|  | rs1018040 | 5.00E-06 |
|  | rs1918172 | 5.00E-06 |
|  | rs17651978 | 6.00E-06 |
|  | rs17367118 | 9.00E-06 |
|  | rs522958 | 1.00E-06 |
|  | rs272000 | 9.00E-06 |
|  | rs2241685 | 8.00E-06 |
|  | rs16928529 | 4.00E-06 |
|  | rs2587695 | 3.00E-07 |
|  | rs2842643 | 3.00E-06 |
|  | rs2199161 | 2.00E-06 |
|  | rs10786284 | 2.00E-06 |
|  | rs10983238 | 1.00E-07 |
|  | rs11646411 | 7.00E-06 |
|  | rs412050 | 6.00E-06 |
|  | rs2281597 | 5.00E-07 |
|  | rs3799977 | 5.00E-06 |
|  | rs469727 | 8.00E-06 |
|  | rs10514604 | 8.00E-07 |
|  | rs2242073 | 8.00E-06 |
|  | rs864643 | 1.00E-08 |
|  | rs7995215 | 1.00E-08 |
|  | rs7175404 | 6.00E-07 |
|  | rs2502731 | 2.00E-06 |
|  | rs1555322 | 4.00E-06 |
|  | rs4964805 | 5.00E-06 |
|  | rs2237349 | 5.00E-06 |
|  | rs2677744 | 1.00E-06 |
|  | rs515910 | 4.00E-06 |
|  | rs220470 | 1.00E-07 |
|  | rs11243897 | 6.00E-08 |
|  | rs7164335 | 1.00E-07 |
|  | rs7463256 | 3.00E-06 |
|  | rs1464807 | 1.00E-06 |
|  | rs12680109 | 7.00E-06 |
|  | rs1027730 | 7.00E-06 |
|  | rs10485813 | 8.00E-06 |
|  | rs11074889 | 7.00E-07 |
|  | rs2823819 | 7.00E-07 |
|  | rs1859156 | 2.00E-06 |
|  | rs10487524 | 9.00E-06 |
|  | rs10011926 | 8.00E-06 |
|  | rs9810857 | 6.00E-06 |
|  | rs4923705 | 2.00E-06 |
|  | rs438259 | 4.00E-06 |
|  | rs2602381 | 4.00E-06 |
|  | rs8074751 | 1.00E-06 |
|  | rs11079828 | 6.54E-06 |
|  | rs3779312 | 8.38E-06 |
|  | rs616668 | 8.62E-06 |
|  | rs42259 | 6.76E-06 |
|  | rs1744062 | 4.16E-06 |
|  | rs10521115 | 8.00E-06 |
|  | rs11642377 | 7.00E-06 |
|  | rs9364220 | 7.00E-06 |
|  | rs9364220 | 6.00E-06 |
|  | rs1822881 | 4.00E-06 |
|  | rs4810796 | 3.00E-06 |
|  | rs4810796 | 3.00E-06 |
|  | rs4708431 | 7.00E-06 |
|  | rs12931939 | 6.00E-06 |
|  | rs12613775 | 4.00E-06 |
|  | rs10180522 | 7.00E-06 |
|  | rs11681930 | 5.00E-06 |
|  | rs2192271 | 1.00E-06 |
|  | rs2119507 | 4.00E-06 |
|  | rs12671878 | 1.00E-06 |
|  | rs13006237 | 9.00E-06 |
|  | rs10153620 | 6.00E-06 |
|  | rs1036736 | 4.00E-06 |
|  | rs12513840 | 7.00E-06 |
|  | rs7201408 | 7.00E-06 |
|  | rs4835929 | 7.00E-06 |
|  | rs2608200 | 6.00E-06 |
|  | rs12919130 | 2.00E-06 |
|  | rs6119285 | 4.00E-06 |
|  | rs10463832 | 5.00E-06 |
|  | rs6758152 | 3.00E-06 |
|  | rs6057659 | 5.00E-06 |
|  | rs17123726 | 5.00E-06 |
|  | rs1515641 | 4.00E-06 |
|  | rs6057648 | 4.00E-06 |
|  | rs7270085 | 4.00E-06 |
|  | rs12926725 | 3.00E-06 |
|  | rs11687420 | 4.00E-06 |
|  | rs6119286 | 4.00E-06 |
|  | rs7448069 | 1.00E-06 |
|  | rs2110267 | 6.00E-06 |
|  | rs6947495 | 1.00E-06 |
|  | rs2110267 | 5.00E-07 |
|  | rs12596252 | 3.00E-06 |
|  | rs1902813 | 3.00E-06 |
|  | rs910191 | 2.00E-06 |
|  | rs11994034 | 7.00E-06 |
|  | rs4458264 | 3.00E-06 |
|  | rs4402823 | 3.00E-06 |
|  | rs13043694 | 3.00E-06 |
|  | rs8123073 | 5.00E-06 |
|  | rs6983777 | 8.00E-06 |
|  | rs910191 | 2.00E-06 |
|  | rs7164923 | 5.00E-06 |
|  | rs4458264 | 3.00E-06 |
|  | rs4402823 | 3.00E-06 |
|  | rs13043694 | 3.00E-06 |
|  | rs11903187 | 4.00E-06 |
|  | rs10193430 | 4.00E-06 |
|  | rs6497436 | 6.00E-06 |
|  | rs12926729 | 7.00E-06 |
|  | rs11647507 | 7.00E-06 |
|  | rs7185264 | 7.00E-06 |
|  | rs12924103 | 7.00E-06 |
|  | rs10521114 | 8.00E-06 |
|  | rs13407123 | 8.00E-06 |
|  | rs7722425 | 5.00E-06 |
|  | rs10463833 | 5.00E-06 |
|  | rs12523164 | 6.00E-06 |
|  | rs6057651 | 4.00E-06 |
|  | rs6057652 | 4.00E-06 |
|  | rs6057652 | 7.00E-06 |
|  | rs6057651 | 7.00E-06 |
|  | rs6453417 | 5.00E-06 |
|  | rs4245040 | 7.00E-06 |
|  | rs17232800 | 3.00E-06 |
|  | rs16828074 | 6.00E-09 |
|  | rs2415545 | 3.80E-06 |
|  | rs2415545 | 1.80E-06 |
|  | rs2415543 | 7.30E-06 |
|  | rs11646443 | 3.80E-07 |
|  | rs12590172 | 2.80E-06 |
|  | rs3814860 | 6.80E-06 |
|  | rs4416909 | 4.50E-06 |
|  | rs1445594 | 3.70E-06 |
|  | rs17696574 | 2.50E-06 |
|  | rs11644983 | 5.40E-06 |
|  | rs1988353 | 1.20E-06 |
|  | rs1897792 | 5.80E-06 |
|  | rs4902569 | 6.20E-06 |
|  | rs4902569 | 5.80E-06 |
|  | rs2038278 | 7.70E-06 |
|  | rs10594 | 2.30E-06 |
|  | rs2232429 | 7.70E-08 |
|  | rs12497166 | 4.95E-06 |
|  | rs9836412 | 4.18E-06 |
|  | rs1019897 | 2.55E-06 |
|  | rs9834616 | 6.25E-06 |
|  | rs17137481 | 9.08E-06 |
|  | rs2856244 | 8.69E-06 |
|  | rs9949006 | 1.38E-06 |

**Supplementary Table S8:** Genes/proteins/molecules from the molecular landscape linked to aggressive behavior through genetic and/or functional evidence.

| **Genes/proteins/molecules** | **Link to aggressive behavior** |
| --- | --- |
| Serotonin | The role of serotonin in aggression has been demonstrated in a wide variety of human and animal studies . |
| Testosterone | Levels of the male hormone testosterone were shown to be correlated with aggression . Altered testosterone-to-cortisol ratio may be associated with aggression in humans . |
| Triiodothyronine | Elevated levels of the active thyroid hormone triiodothyronine have been associated with conduct disorder and criminal behavior . |
| Growth hormone | *GHRH* knock- out mice with growth hormone (GH) deficiency show reduced aggressive behavior which can be normalized by GH replacement . |
| Retinoic acid | Chronic administration of synthetic retinoid acid to rats reduced aggression in the resident-intruder paradigm . |
| Nerve growth factor | NGF is thought to be involved in aggression and alcohol dependence and changes in levels of nerve growth factor have been observed in rodents following aggressive intermale interactions . |
| *NTRK1* | Fighting in male mice potentiates mRNA for the high affinitiy nerve growth factor receptor TrkA (encoded by the *NTRK1* [gene](http://en.wikipedia.org/wiki/Gene) in humans) in the subventricular zone and hippocampus . |
| Protein Kinase A | Coronin 1 deficiency in mouse and human causes severe neurobehavioral defects, including increased aggression and social deficits, through modulation of cAMP/Protein Kinase A Signaling . |
| cAMP | Coronin 1 deficiency in mouse and human causes severe neurobehavioral defects, including increased aggression and social deficits, through modulation of cAMP/Protein Kinase A Signaling . |
| *AR* | The androgen receptor (*AR*) CAG repeat motif has been associated with aggressive and violent behavior in men . |


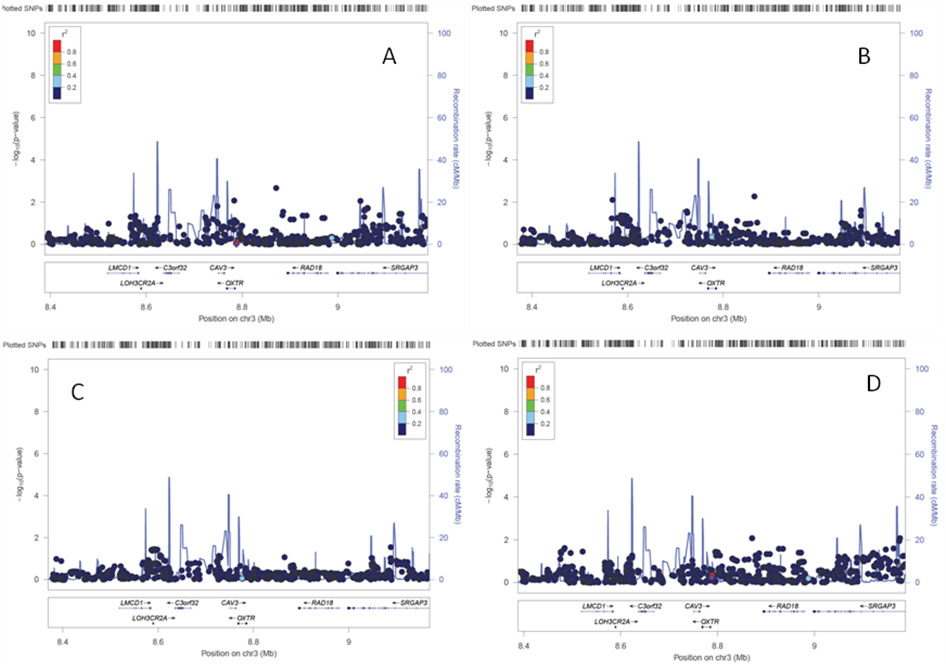


**Supplementary Figure S1**. Outcome of the association analysis of the two ODD subtypes and the two ODD dimensions for all SNPs located in the *OXTR* region. **A.** P1 (defiant vindictive), **B.** P2 (irritability), **C.** P3 (0 representing ‘low OPP/moderate OPP’ and 1 representing ‘irritable OPP/severe OPP’) and **D.** P4 (0 representing ‘low OPP/moderate OPP/irritable OPP’ and 1 representing ‘severe OPP’). SNP rs1488467 and linked SNPs are depicted in color.


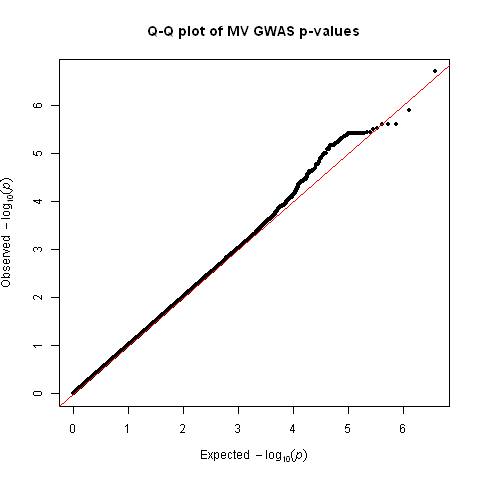


**Supplementary Figure S2.** Quantile quantile plot for the multivariate GWAS.


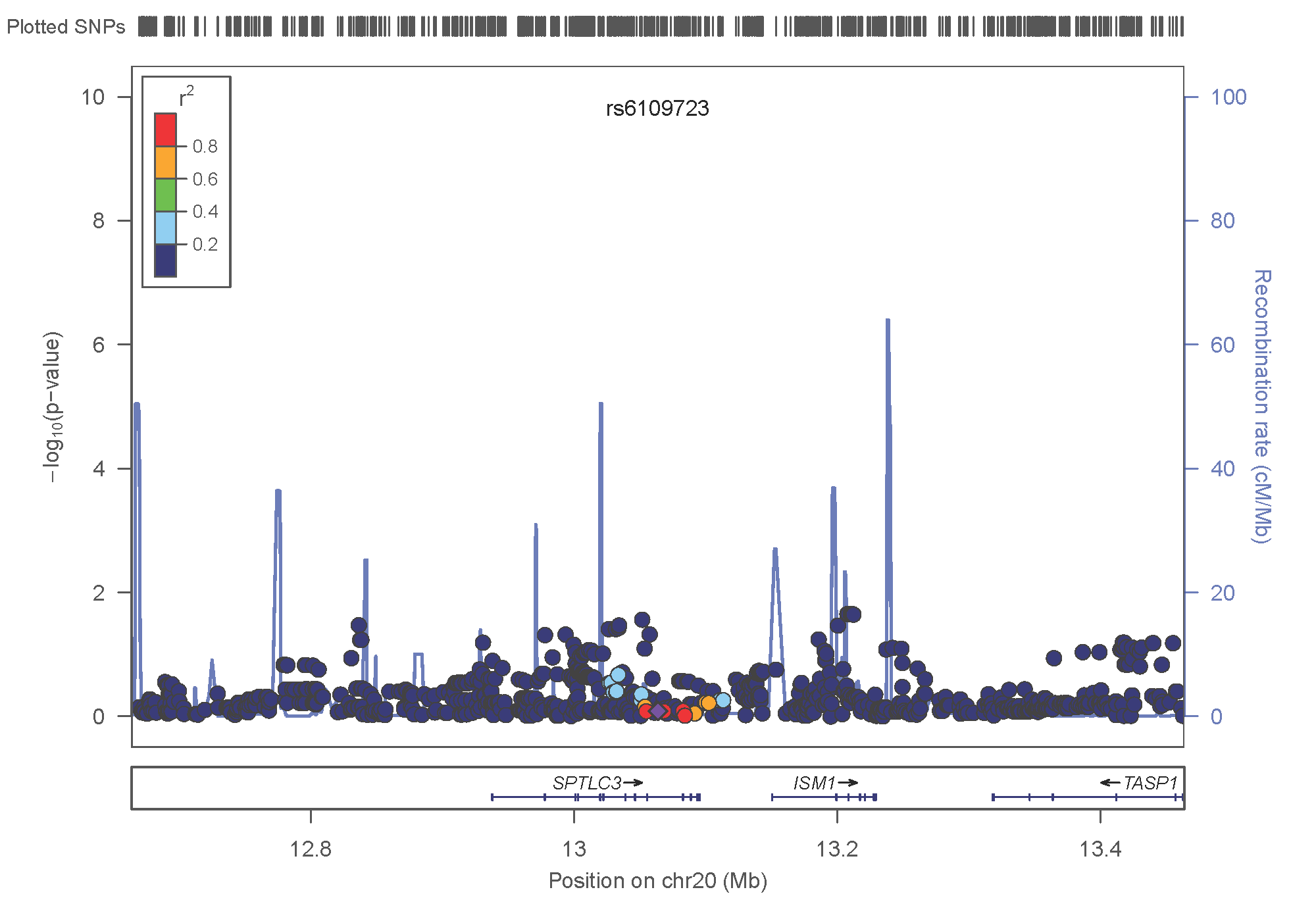

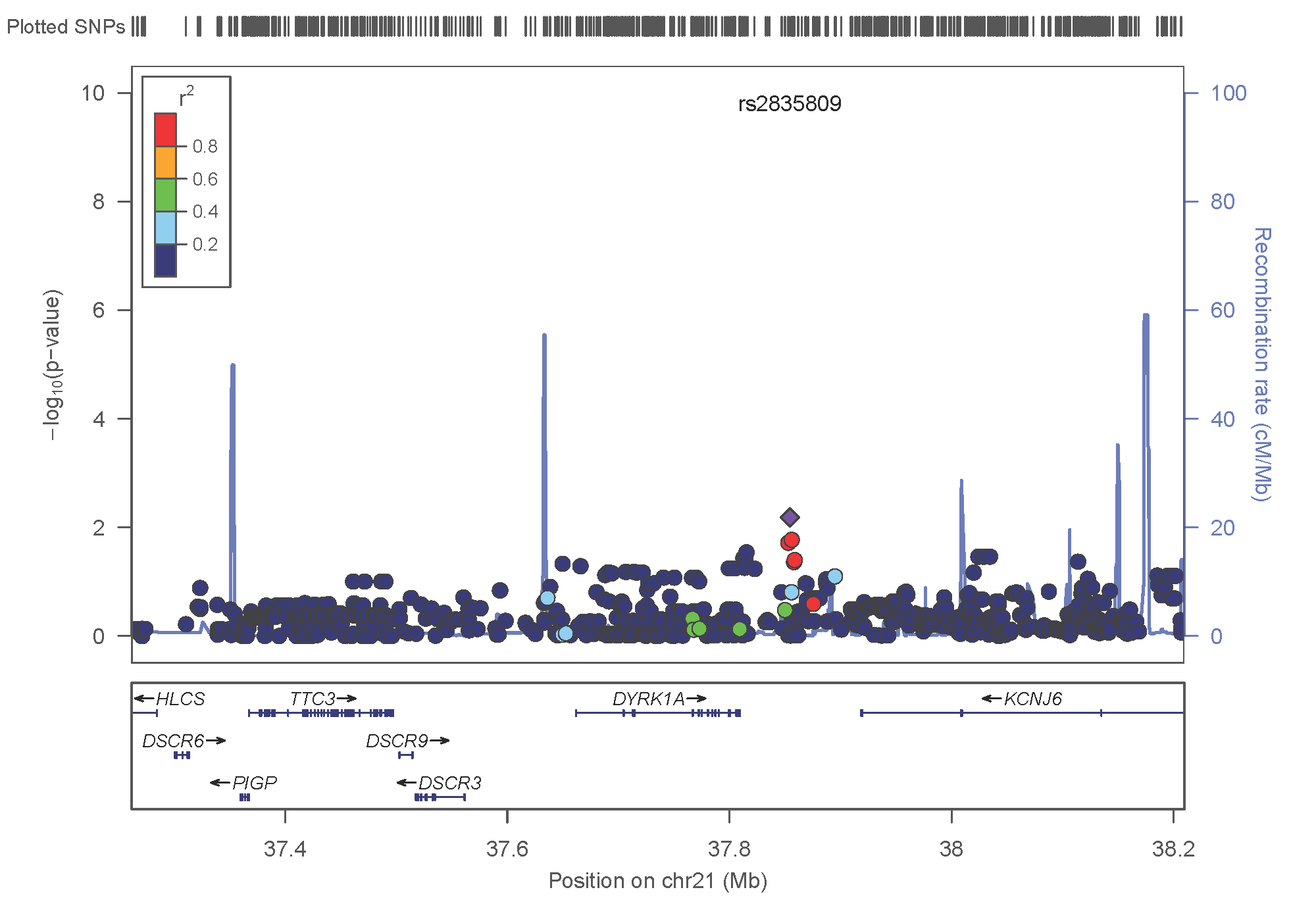

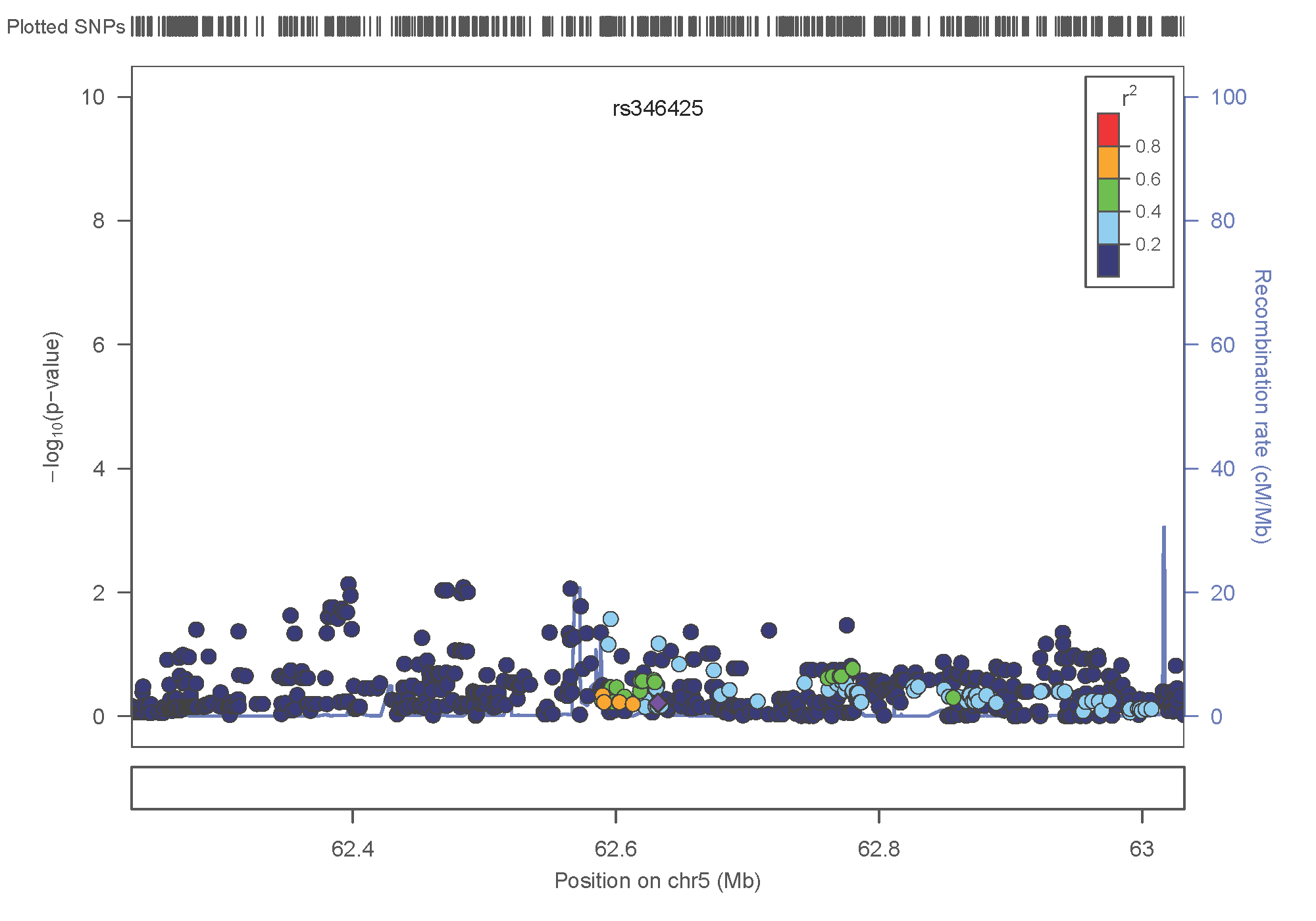

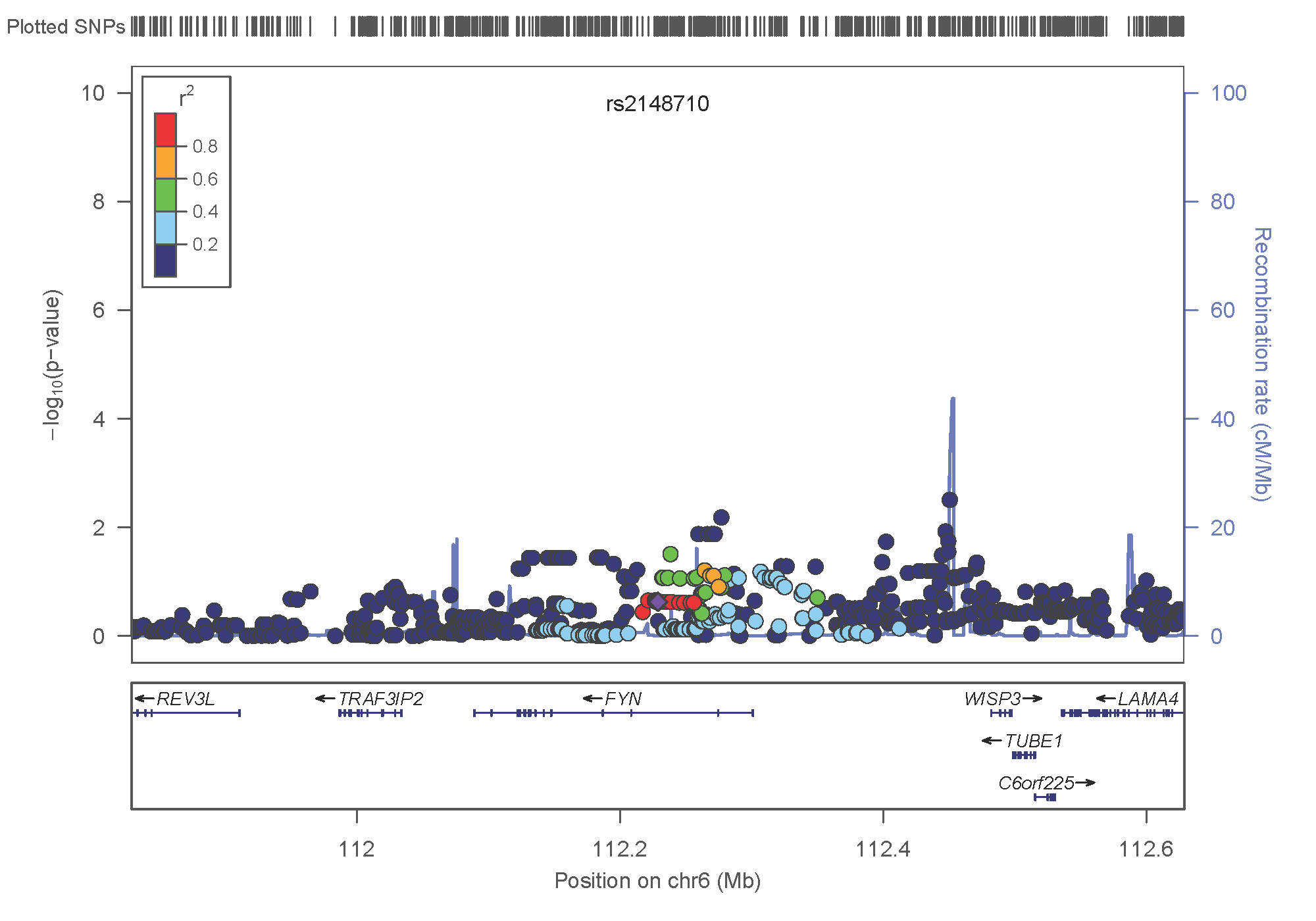

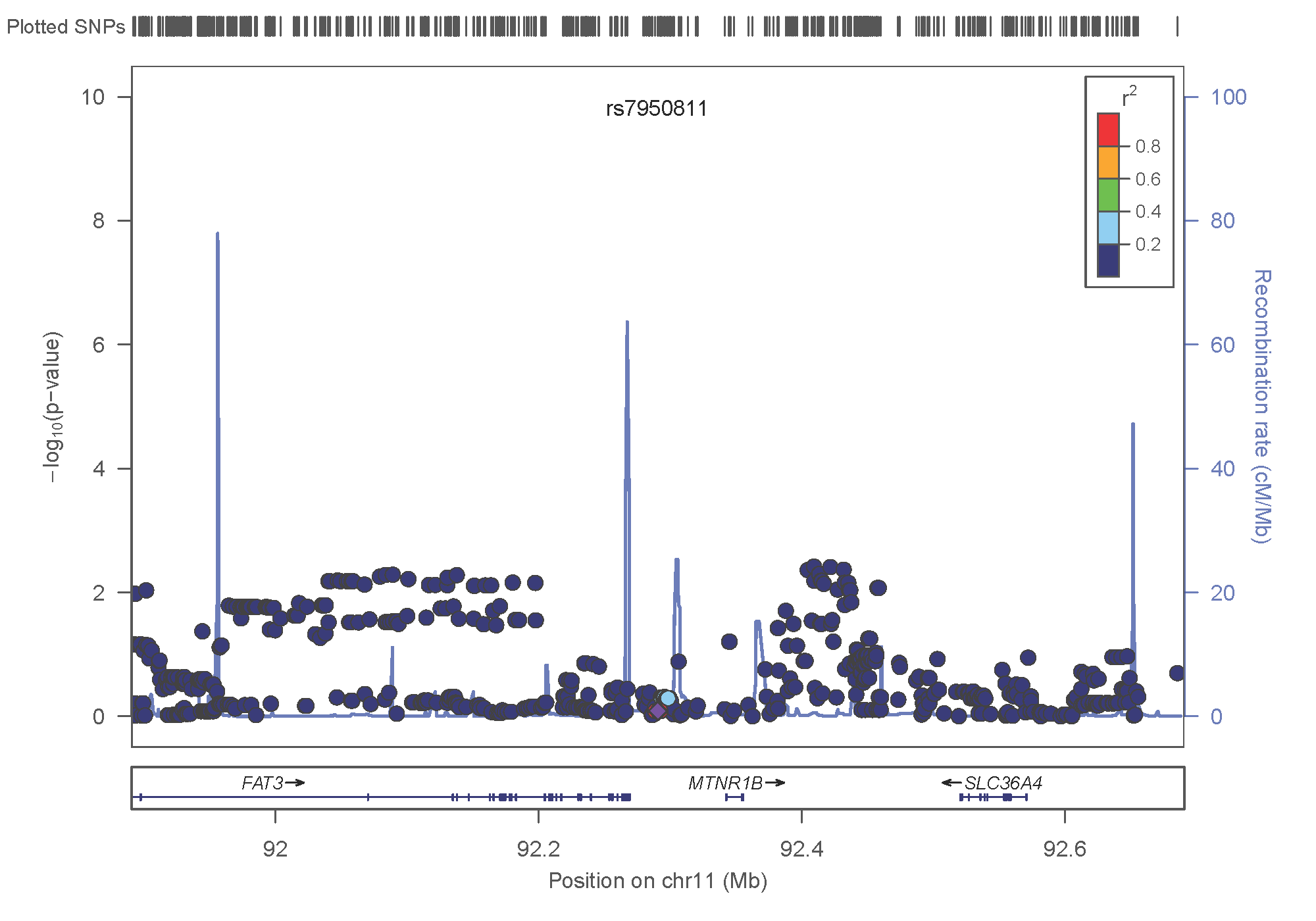

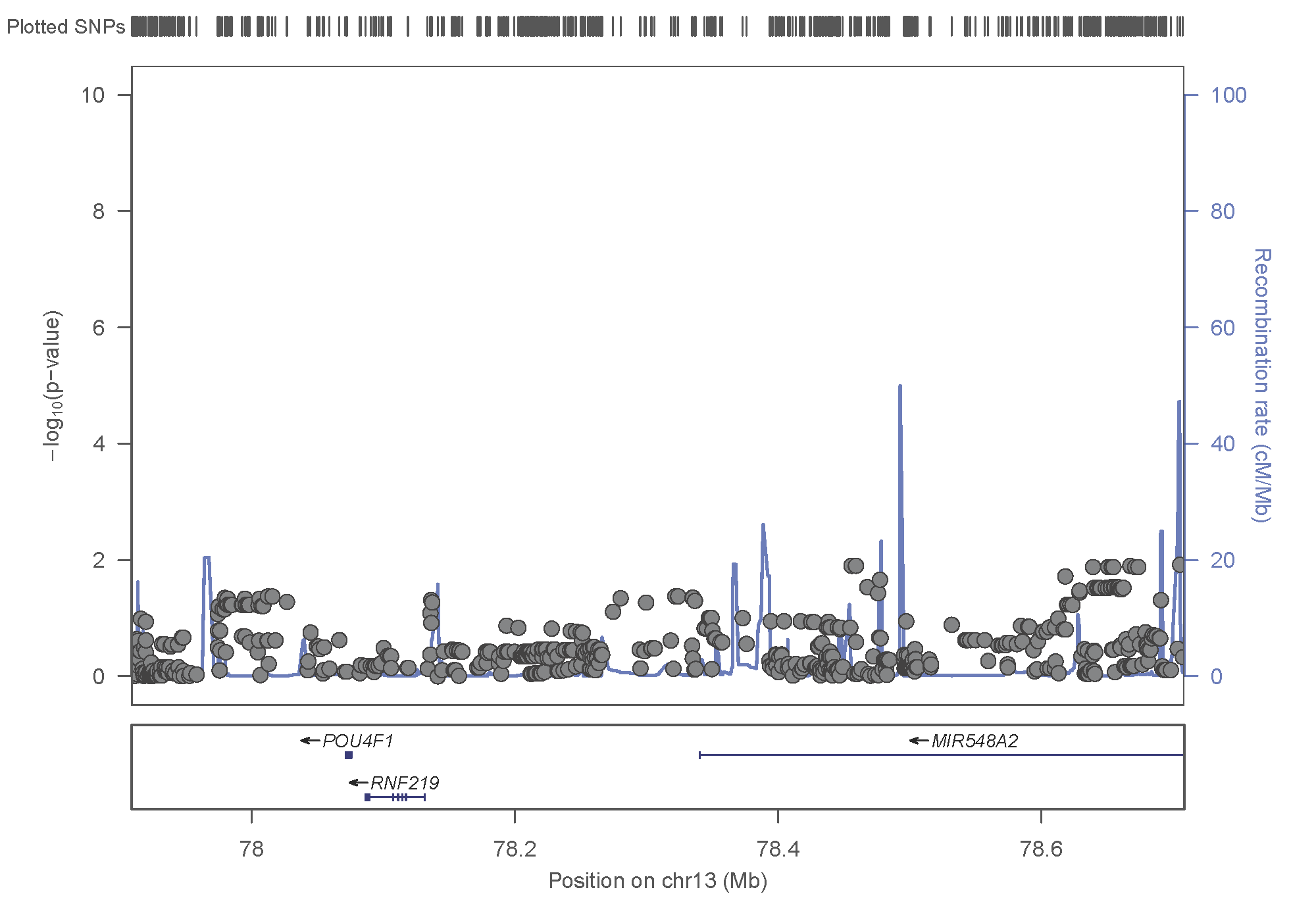

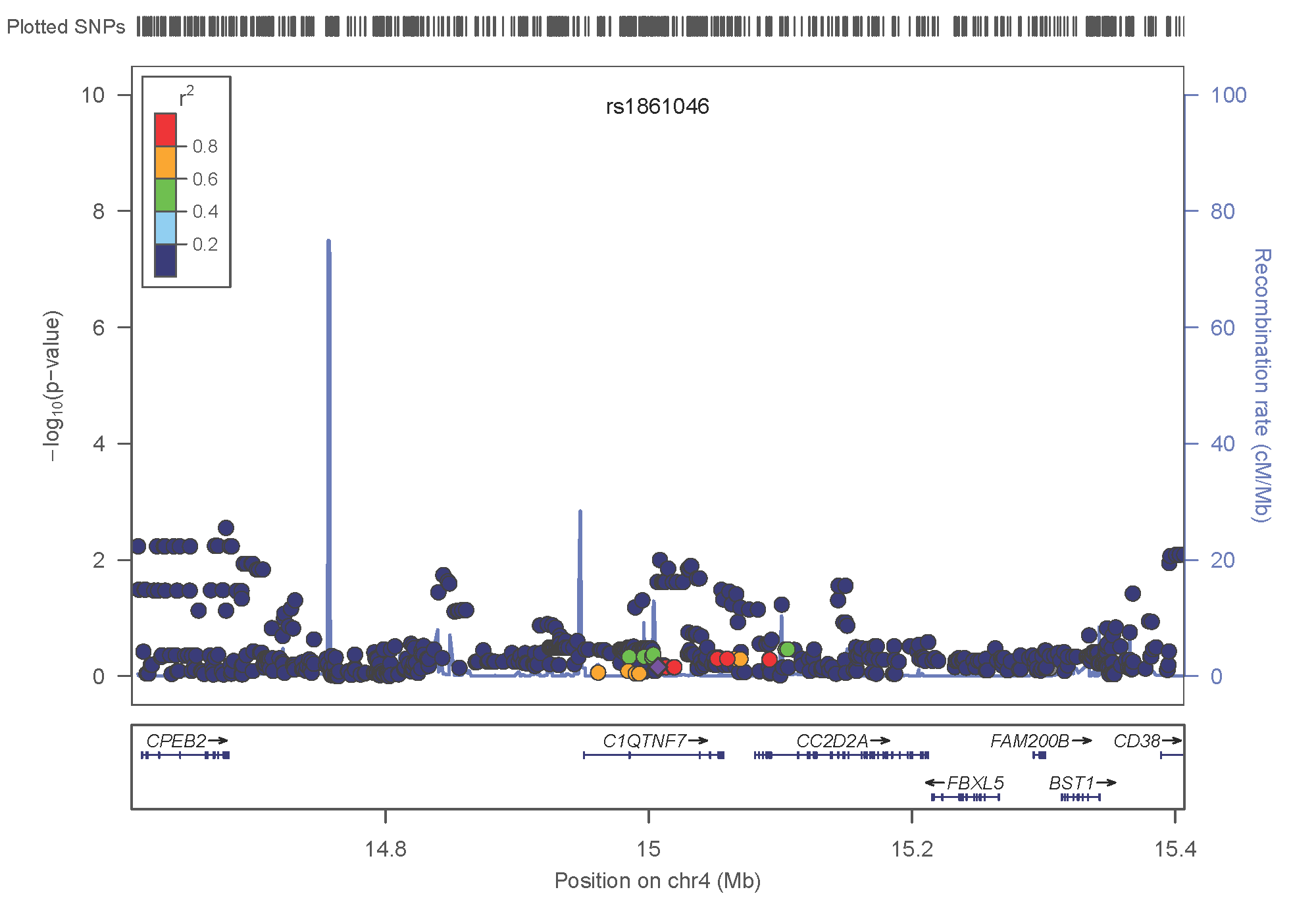


**A**

**A**

**B**

**C**

**C**

**C**

**D**

**Supplementary Figure S3:** Comparison with results of previous aggression related GWAS. Top findings including a 400 kb surrounding region are shown. **A**: GWAS of adult antisocial behavior by : Top SNP rs346425 on chromosome 5 and top gene DYRK1A on chromosome 21. **B**: GWAS of proneness to anger by : Potentially significant SNP rs2148710 on chromosome 6. **C**: GWAS of CD symptom count by : Four genome-wide significant SNPs; rs7950811 on chromosome 11, rs11838918 and rs16891867 (not shown here) on chromosome 13 and rs1861046 on chromosome 4. **D**: GWAS of aggressiveness-hostility by : Top SNP rs17190927 on chromosome 20 (rs6109723 (r^2^=1) plotted).

**Supplemental description of the molecular ODD landscape and legend (Figure 3)**

The ODD candidate gene/proteins that were implicated by our GWAS (**Supplementary Table S4**) and the other ODD/aggressive behavior candidate genes/proteins/molecules (**Supplementary Table S7**) are indicated in bold.

Signaling through the landscape can be initiated at the neuronal cell membrane, where the binding of ligands from the extracellular matrix to their receptors leads to the modulation of downstream molecular cascades in the cytoplasm, cytoskeleton and nucleus that are involved in regulating neurite outgrowth. Furthermore, modulation of the neuronal extracellular matrix is necessary for the neurite to grow in a certain direction. The main signaling cascade in the landscape centers around β-catenin (CTNNB), a protein that has a dual function as a peripheral membrane/cytoplasmic protein and nuclear transcription factor and regulates neurite outgrowth .

CDH2 and **CDH4**, two proteins that mediate cell adhesion and regulate neurite outgrowth , are anchored in the neuronal cell membrane through forming a functional complex with each other and the peripheral membrane proteins CTNNB and CTNND1 . The activity of CTNND1 is regulated by the neurite outgrowth-implicated kinase **PAK7**  and both CTNNB and CTNND1 can function as a transcription factor (see below). Moreover, **SLIT1** binding to the ROBO1 axon guidance receptor inactivates CDH2, which leads to CTNNB being released from CDH2 and targeted to the cytoplasm and subsequently the nucleus . Through degrading CDH2, the extracellular metalloprotease **ADAM10** also promotes the translocation of CTNNB from the peripheral membrane to the nucleus . In addition, **ADAM10** is involved in regulating the expression of MAGI2 , a scaffolding protein that binds and regulates the activity of CTNNB and that has been implicated in neurite outgrowth . MAGI2 also binds and functionally modulates the neuronal membrane receptor **LPHN2**  and, through binding and regulating CTNNB, it is functionally linked to **TRPC4**, a calcium channel that directly binds and interacts with CTNNB and stimulates neurite outgrowth , and the CDH2-**CDH4**-CTNND1 complex (see above; not shown). Furthermore, extracellular **SFRP4** regulates the activity and localization of CTNNB while **PCDH20**, a membrane protein that like CDH2 and **CDH4** mediates (neuronal) cell adhesion , inhibits the cytoplasmic/nuclear translocation and function of CTNNB and the **STK39** kinase binds and functionally interacts with CTNNB .

After having translocated to the nucleus, CTNNB functions as a transcription factor. The transcriptional activity of CTNNB is inhibited by two other transcription factors, i.e., **SOX5** and the **androgen receptor (AR)** bound to and activated by **testosterone ,** the male sex hormone that positively regulates neurite outgrowth , and is produced by a number of enzymes in the endoplasmic reticulum, including **HSD17B3** . Further, the expression of CTNNB is upregulated by nuclear and **PAK7**-activated (see above) CTNND1 and **RUNX1T1** , a transcription factor that binds and activates **ZBTB16**, another transcription factor . In turn, **ZBTB16** binds and inhibits the transcriptional activity of retinoic acid receptor alpha (RARA) , a nuclear receptor that, when bound and activated by **retinoic acid,** forms a functional complex with CTNNB and downregulates the expression of the **retinoic acid receptor beta (RARB)** , a transcription factor with an established role in stimulating neurite outgrowth . In addition, **C1D** binds and modulates the transcriptional activity of THRB , a nuclear receptor that when bound and activated by **triiodothyronine (T3)** - the active thyroid hormone that promotes neurite outgrowth - upregulates the expression of CTNNB .

When activated, CTNNB (up)regulates the expression of the **growth hormone (GH) receptor** (**GHR)** , **PDE1C** and **MMP7** while it downregulates the expression of the **nerve growth factor (NGF)** receptor (NGFR) . The **GH-GHR** complex is involved in inducing neurite outgrowth while **NGF** regulates this process through binding specifically to NGFR or **NTRK1** , with the **NGF-NTRK1** complex also being bound and functionally modulated by the adaptor protein **SHC3** . In addition, **NGF** promotes neurite outgrowth through upregulating the expression of the sodium channel **ASIC2** . Furthermore, **PDE1C** is a cytoplasmic enzyme that degrades **cyclic AMP (cAMP)** , a second messenger molecule that regulates many physiological processes through activating **protein kinase A (PKA)** , which itself is an important regulator of neurite outgrowth . **PKA** is activated through **serotonin** binding to the HTR7 receptor , which is directly bound and modulated by **RHOBTB3** , and activates/stabilizes CTNNB and CDC42 , an important mediator of directed neurite outgrowth that binds and activates **PAK7** . CDC42 is also activated downstream of **TENM4**, a membrane protein that promotes neurite outgrowth .

Furthermore, **PKA** is directly involved in remodelling the neuronal cytoskeleton - which is essential for neurite outgrowth to take place - through regulating the stability of actin filaments that together with microtubules form the cytoskeleton , and both **ABLIM2** and **AFAP1** have a similar effect through directly binding and affecting the stability of actin filaments in the (neuronal) cytoskeleton.

As already indicated above and in addition to cytoskeletal remodelling, the neuronal extracellular matrix has to be modulated for the neurite to grow in a certain direction . In this respect, the brain-expressed metalloproteinases **ADAM10, ADAM12**, **ADAMTSL3** and **MMP7** - which are also upregulated by CTNNB (see above) -regulate neurite outgrowth through degrading the neuronal extracellular matrix . Moreover, **MMP7** is involved in upregulating the expression of **ADAM12** and **ADAMTSL3** degrades fibrillin-1 (FBN1) which in turn regulates the expression of **SFRP4** . Furthermore, in addition to the interactions already described above, **ADAM10** regulates the expression of **SOSTDC1** , a brain-expressed extracellular matrix protein . Lastly, **ISPD** is an extracellular enzyme that, similar to the **SLIT1**-ROBO1 complex (see above), is involved in regulating axon guidance and hence neurite outgrowth .

**Additional References in Supplement**

Agiostratidou, G, Li, M, Suyama, K, Badano, I, Keren, R, Chung, S, Anzovino, A, Hulit, J, Qian, B, Bouzahzah, B, Eugenin, E, Loudig, O, Phillips, GR, Locker, J, Hazan, RB. 2009. Loss of retinal cadherin facilitates mammary tumor progression and metastasis. Cancer Res, 69: 5030-5038.

Aglah, C, Gordon, T, Posse de Chaves, EI. 2008. cAMP promotes neurite outgrowth and extension through protein kinase A but independently of Erk activation in cultured rat motoneurons. Neuropharmacology, 55: 8-17.

Agudo, M, Yip, P, Davies, M, Bradbury, E, Doherty, P, McMahon, S, Maden, M, Corcoran, JP. 2010. A retinoic acid receptor beta agonist (CD2019) overcomes inhibition of axonal outgrowth via phosphoinositide 3-kinase signalling in the injured adult spinal cord. Neurobiol Dis, 37: 147-155.

Alcocer-Gomez, E, de Miguel, M, Casas-Barquero, N, Nunez-Vasco, J, Sanchez-Alcazar, JA, Fernandez-Rodriguez, A, Cordero, MD. 2014. NLRP3 inflammasome is activated in mononuclear blood cells from patients with major depressive disorder. Brain Behav Immun, 36: 111-117.

Alliey-Rodriguez, N, Zhang, D, Badner, JA, Lahey, BB, Zhang, X, Dinwiddie, S, Romanos, B, Plenys, N, Liu, C, Gershon, ES. 2011. Genome-wide association study of personality traits in bipolar patients. Psychiatr Genet, 21: 190-194.

Anholt, RR, Mackay, TF. 2012. Genetics of aggression. Annu Rev Genet, 46: 145-164.

Arodin, L, Lamparter, H, Karlsson, H, Nennesmo, I, Bjornstedt, M, Schroder, J, Fernandes, AP. 2014. Alteration of thioredoxin and glutaredoxin in the progression of Alzheimer's disease. J Alzheimers Dis, 39: 787-797.

Barrientos, T, Frank, D, Kuwahara, K, Bezprozvannaya, S, Pipes, GC, Bassel-Duby, R, Richardson, JA, Katus, HA, Olson, EN, Frey, N. 2007. Two novel members of the ABLIM protein family, ABLIM-2 and -3, associate with STARS and directly bind F-actin. J Biol Chem, 282: 8393-8403.

Baudet, ML, Hassanali, Z, Sawicki, G, List, EO, Kopchick, JJ, Harvey, S. 2008. Growth hormone action in the developing neural retina: a proteomic analysis. Proteomics, 8: 389-401.

Bayle, J, Fitch, J, Jacobsen, K, Kumar, R, Lafyatis, R, Lemaire, R. 2008. Increased expression of Wnt2 and SFRP4 in Tsk mouse skin: role of Wnt signaling in altered dermal fibrillin deposition and systemic sclerosis. J Invest Dermatol, 128: 871-881.

Bergen, SE, O'Dushlaine, CT, Ripke, S, Lee, PH, Ruderfer, DM, Akterin, S, Moran, JL, Chambert, KD, Handsaker, RE, Backlund, L, Osby, U, McCarroll, S, Landen, M, Scolnick, EM, Magnusson, PK, Lichtenstein, P, Hultman, CM, Purcell, SM, Sklar, P, Sullivan, PF. 2012. Genome-wide association study in a Swedish population yields support for greater CNV and MHC involvement in schizophrenia compared with bipolar disorder. Mol Psychiatry, 17: 880-886.

Berndt, T, Craig, TA, Bowe, AE, Vassiliadis, J, Reczek, D, Finnegan, R, Jan De Beur, SM, Schiavi, SC, Kumar, R. 2003. Secreted frizzled-related protein 4 is a potent tumor-derived phosphaturic agent. J Clin Invest, 112: 785-794.

Bhardwaj, D, Nager, M, Camats, J, David, M, Benguria, A, Dopazo, A, Canti, C, Herreros, J. 2013. Chemokines induce axon outgrowth downstream of Hepatocyte Growth Factor and TCF/beta-catenin signaling. Front Cell Neurosci, 7: 52.

Brown, MD, Cornejo, BJ, Kuhn, TB, Bamburg, JR. 2000. Cdc42 stimulates neurite outgrowth and formation of growth cone filopodia and lamellipodia. J Neurobiol, 43: 352-364.

Burden-Gulley, SM, Gates, TJ, Craig, SE, Gupta, M, Brady-Kalnay, SM. 2010. Stimulation of N-cadherin-dependent neurite outgrowth by small molecule peptide mimetic agonists of the N-cadherin HAV motif. Peptides, 31: 842-849.

Chen, N, Furuya, S, Shinoda, Y, Yumoto, M, Ohtake, A, Sato, K, Doi, H, Hashimoto, Y, Kudo, Y, Higashi, H. 2003. Extracellular carbohydrate-signal triggering cAMP-dependent protein kinase-dependent neuronal actin-reorganization. Neuroscience, 122: 985-995.

Craig, IW, Halton, KE. 2009. Genetics of human aggressive behaviour. Hum Genet, 126: 101-113.

Dan, C, Nath, N, Liberto, M, Minden, A. 2002. PAK5, a new brain-specific kinase, promotes neurite outgrowth in N1E-115 cells. Mol Cell Biol, 22: 567-577.

Dey, N, Young, B, Abramovitz, M, Bouzyk, M, Barwick, B, De, P, Leyland-Jones, B. 2013. Differential activation of Wnt-beta-catenin pathway in triple negative breast cancer increases MMP7 in a PTEN dependent manner. PLoS One, 8: e77425.

Dick, DM, Aliev, F, Krueger, RF, Edwards, A, Agrawal, A, Lynskey, M, Lin, P, Schuckit, M, Hesselbrock, V, Nurnberger, J, Jr., Almasy, L, Porjesz, B, Edenberg, HJ, Bucholz, K, Kramer, J, Kuperman, S, Bierut, L. 2011. Genome-wide association study of conduct disorder symptomatology. Mol Psychiatry, 16: 800-808.

Dow, DJ, Huxley-Jones, J, Hall, JM, Francks, C, Maycox, PR, Kew, JN, Gloger, IS, Mehta, NA, Kelly, FM, Muglia, P, Breen, G, Jugurnauth, S, Pederoso, I, St Clair, D, Rujescu, D, Barnes, MR. 2011. ADAMTSL3 as a candidate gene for schizophrenia: gene sequencing and ultra-high density association analysis by imputation. Schizophr Res, 127: 28-34.

Drummond, HA, Furtado, MM, Myers, S, Grifoni, S, Parker, KA, Hoover, A, Stec, DE. 2006. ENaC proteins are required for NGF-induced neurite growth. Am J Physiol Cell Physiol, 290: C404-410.

Easwaran, V, Pishvaian, M, Salimuddin, Byers, S. 1999. Cross-regulation of beta-catenin-LEF/TCF and retinoid signaling pathways. Curr Biol, 9: 1415-1418.

Ebejer, JL, Duffy, DL, van der Werf, J, Wright, MJ, Montgomery, G, Gillespie, NA, Hickie, IB, Martin, NG, Medland, SE. 2013. Genome-wide association study of inattention and hyperactivity-impulsivity measured as quantitative traits. Twin Res Hum Genet, 16: 560-574.

Estrada, M, Uhlen, P, Ehrlich, BE. 2006. Ca2+ oscillations induced by testosterone enhance neurite outgrowth. J Cell Sci, 119: 733-743.

Farkas, N, Lendeckel, U, Dobrowolny, H, Funke, S, Steiner, J, Keilhoff, G, Schmitt, A, Bogerts, B, Bernstein, H-G. 2010. Reduced density of ADAM 12-immunoreactive oligodendrocytes in the anterior cingulate white matter of patients with schizophrenia. World J Biol Psychiatry, 11: 556-566.

Fiore, M, Amendola, T, Triaca, V, Alleva, E, Aloe, L. 2005. Fighting in the aged male mouse increases the expression of TrkA and TrkB in the subventricular zone and in the hippocampus. Behav Brain Res, 157: 351-362.

Garshasbi, M, Motazacker, MM, Kahrizi, K, Behjati, F, Abedini, SS, Nieh, SE, Firouzabadi, SG, Becker, C, Ruschendorf, F, Nurnberg, P, Tzschach, A, Vazifehmand, R, Erdogan, F, Ullmann, R, Lenzner, S, Kuss, AW, Ropers, HH, Najmabadi, H. 2006. SNP array-based homozygosity mapping reveals MCPH1 deletion in family with autosomal recessive mental retardation and mild microcephaly. Hum Genet, 118: 708-715.

Gavard, J, Marthiens, V, Monnet, C, Lambert, M, Mege, RM. 2004. N-cadherin activation substitutes for the cell contact control in cell cycle arrest and myogenic differentiation: involvement of p120 and beta-catenin. J Biol Chem, 279: 36795-36802.

Genomics, C-DGotP. 2013. Identification of risk loci with shared effects on five major psychiatric disorders: a genome-wide analysis. Lancet, 381: 1371-1379.

Gervasi, N, Hepp, R, Tricoire, L, Zhang, J, Lambolez, B, Paupardin-Tritsch, D, Vincent, P. 2007. Dynamics of protein kinase A signaling at the membrane, in the cytosol, and in the nucleus of neurons in mouse brain slices. J Neurosci, 27: 2744-2750.

Graziani, A, Poteser, M, Heupel, WM, Schleifer, H, Krenn, M, Drenckhahn, D, Romanin, C, Baumgartner, W, Groschner, K. 2010. Cell-cell contact formation governs Ca2+ signaling by TRPC4 in the vascular endothelium: evidence for a regulatory TRPC4-beta-catenin interaction. J Biol Chem, 285: 4213-4223.

Gregersen, NO, Buttenschon, HN, Hedemand, A, Dahl, HA, Kristensen, AS, Clementsen, B, Woldbye, DPD, Koefoed, P, Erhardt, A, Kruse, TA, Wang, AG, Borglum, AD, Mors, O. 2014. Are TMEM genes potential candidate genes for panic disorder? Psychiatr Genet, 24: 37-41.

Grigoryan, T, Stein, S, Qi, J, Wende, H, Garratt, AN, Nave, KA, Birchmeier, C, Birchmeier, W. 2013. Wnt/Rspondin/beta-catenin signals control axonal sorting and lineage progression in Schwann cell development. Proc Natl Acad Sci U S A, 110: 18174-18179.

Grimbly, C, Martin, B, Karpinski, E, Harvey, S. 2009. Growth hormone production and action in N1E-115 neuroblastoma cells. J Mol Neurosci, 39: 117-124.

Haller, J. 2012. The neurobiology of abnormal manifestations of aggression-A review of hypothalamic mechanisms in cats, rodents, and humans. Brain Res Bull, 93: 97-109.

Hansen, SM, Berezin, V, Bock, E. 2008. Signaling mechanisms of neurite outgrowth induced by the cell adhesion molecules NCAM and N-cadherin. Cell Mol Life Sci, 65: 3809-3821.

Harold, D, Jehu, L, Turic, D, Hollingworth, P, Moore, P, Summerhayes, P, Moskvina, V, Foy, C, Archer, N, Hamilton, BA, Lovestone, S, Powell, J, Brayne, C, Rubinsztein, DC, Jones, L, O'Donovan, MC, Owen, MJ, Williams, J. 2007. Interaction between the ADAM12 and SH3MD1 genes may confer susceptibility to late-onset Alzheimer's disease. Am J Med Genet B Neuropsychiatr Genet, 144B: 448-452.

Hazan, RB, Norton, L. 1998. The epidermal growth factor receptor modulates the interaction of E-cadherin with the actin cytoskeleton. J Biol Chem, 273: 9078-9084.

Hinney, A, Scherag, A, Jarick, I, Albayrak, O, Putter, C, Pechlivanis, S, Dauvermann, MR, Beck, S, Weber, H, Scherag, S, Nguyen, TT, Volckmar, AL, Knoll, N, Faraone, SV, Neale, BM, Franke, B, Cichon, S, Hoffmann, P, Nothen, MM, Schreiber, S, Jockel, KH, Wichmann, HE, Freitag, C, Lempp, T, Meyer, J, Gilsbach, S, Herpertz-Dahlmann, B, Sinzig, J, Lehmkuhl, G, Renner, TJ, Warnke, A, Romanos, M, Lesch, KP, Reif, A, Schimmelmann, BG, Hebebrand, J. 2011. Genome-wide association study in German patients with attention deficit/hyperactivity disorder. Am J Med Genet B Neuropsychiatr Genet, 156b: 888-897.

Hino, S, Tanji, C, Nakayama, KI, Kikuchi, A. 2005. Phosphorylation of beta-catenin by cyclic AMP-dependent protein kinase stabilizes beta-catenin through inhibition of its ubiquitination. Mol Cell Biol, 25: 9063-9072.

Hoecker, J, Liffert, R, Burch, P, Wehlauch, R, Gademann, K. 2013. Caged retinoids as photoinducible activators: implications for cell differentiation and neurite outgrowth. Org Biomol Chem, 11: 3314-3321.

Hosseini, MM, Tonekaboni, SH, Papari, E, Bahman, I, Behjati, F, Kahrizi, K, Najmabadi, H. 2012. A novel mutation in MCPH1 gene in an Iranian family with primary microcephaly. J Pak Med Assoc, 62: 1244-1247.

Huang, EJ, Reichardt, LF. 2001. Neurotrophins: roles in neuronal development and function. Annu Rev Neurosci, 24: 677-736.

Huang, T-L, Sung, M-L, Chen, T-Y. 2014. 2D-DIGE proteome analysis on the platelet proteins of patients with major depression. Proteome Sci, 12: 1-1.

Hurd, PL, Vaillancourt, KL, Dinsdale, NL. 2011. Aggression, digit ratio and variation in androgen receptor and monoamine oxidase a genes in men. Behav Genet, 41: 543-556.

International Parkinson Disease Genomics, C, Nalls, MA, Plagnol, V, Hernandez, DG, Sharma, M, Sheerin, UM, Saad, M, Simon-Sanchez, J, Schulte, C, Lesage, S, Sveinbjornsdottir, S, Stefansson, K, Martinez, M, Hardy, J, Heutink, P, Brice, A, Gasser, T, Singleton, AB, Wood, NW. 2011. Imputation of sequence variants for identification of genetic risks for Parkinson's disease: a meta-analysis of genome-wide association studies. Lancet, 377: 641-649.

Irish Schizophrenia Genomics, C, the Wellcome Trust Case Control, C. 2012. Genome-wide association study implicates HLA-C*01:02 as a risk factor at the major histocompatibility complex locus in schizophrenia. Biol Psychiatry, 72: 620-628.

Ishiyama, N, Lee, SH, Liu, S, Li, GY, Smith, MJ, Reichardt, LF, Ikura, M. 2010. Dynamic and static interactions between p120 catenin and E-cadherin regulate the stability of cell-cell adhesion. Cell, 141: 117-128.

Jayachandran, R, Liu, X, Bosedasgupta, S, Muller, P, Zhang, CL, Moshous, D, Studer, V, Schneider, J, Genoud, C, Fossoud, C, Gambino, F, Khelfaoui, M, Muller, C, Bartholdi, D, Rossez, H, Stiess, M, Houbaert, X, Jaussi, R, Frey, D, Kammerer, RA, Deupi, X, de Villartay, JP, Luthi, A, Humeau, Y, Pieters, J. 2014. Coronin 1 regulates cognition and behavior through modulation of cAMP/protein kinase A signaling. PLoS Biol, 12: e1001820.

Jones, DC, Kuhar, MJ. 2006. Cocaine-amphetamine-regulated transcript expression in the rat nucleus accumbens is regulated by adenylyl cyclase and the cyclic adenosine 5'-monophosphate/protein kinase a second messenger system. J Pharmacol Exp Ther, 317: 454-461.

Juliano, RL. 2002. Signal transduction by cell adhesion receptors and the cytoskeleton: functions of integrins, cadherins, selectins, and immunoglobulin-superfamily members. Annu Rev Pharmacol Toxicol, 42: 283-323.

Kao, HT, Song, HJ, Porton, B, Ming, GL, Hoh, J, Abraham, M, Czernik, AJ, Pieribone, VA, Poo, MM, Greengard, P. 2002. A protein kinase A-dependent molecular switch in synapsins regulates neurite outgrowth. Nat Neurosci, 5: 431-437.

Kim, M, Suh, J, Romano, D, Truong, MH, Mullin, K, Hooli, B, Norton, D, Tesco, G, Elliott, K, Wagner, SL, Moir, RD, Becker, KD, Tanzi, RE. 2009. Potential late-onset Alzheimer's disease-associated mutations in the ADAM10 gene attenuate {alpha}-secretase activity. Hum Mol Genet, 18: 3987-3996.

Klimov, E, Rud'ko, O, Rakhmanaliev, E, Sulimova, G. 2005. Genomic organisation and tissue specific expression of ABLIM2 gene in human, mouse and rat. Biochim Biophys Acta, 1730: 1-9.

Kohutek, ZA, diPierro, CG, Redpath, GT, Hussaini, IM. 2009. ADAM-10-mediated N-cadherin cleavage is protein kinase C-alpha dependent and promotes glioblastoma cell migration. J Neurosci, 29: 4605-4615.

Kurian, SM, Le-Niculescu, H, Patel, SD, Bertram, D, Davis, J, Dike, C, Yehyawi, N, Lysaker, P, Dustin, J, Caligiuri, M, Lohr, J, Lahiri, DK, Nurnberger, JI, Jr., Faraone, SV, Geyer, MA, Tsuang, MT, Schork, NJ, Salomon, DR, Niculescu, AB. 2011. Identification of blood biomarkers for psychosis using convergent functional genomics. Mol Psychiatry, 16: 37-58.

Lamb, AN, Rosenfeld, JA, Neill, NJ, Talkowski, ME, Blumenthal, I, Girirajan, S, Keelean-Fuller, D, Fan, Z, Pouncey, J, Stevens, C, Mackay-Loder, L, Terespolsky, D, Bader, PI, Rosenbaum, K, Vallee, SE, Moeschler, JB, Ladda, R, Sell, S, Martin, J, Ryan, S, Jones, MC, Moran, R, Shealy, A, Madan-Khetarpal, S, McConnell, J, Surti, U, Delahaye, A, Heron-Longe, B, Pipiras, E, Benzacken, B, Passemard, S, Verloes, A, Isidor, B, Le Caignec, C, Glew, GM, Opheim, KE, Descartes, M, Eichler, EE, Morton, CC, Gusella, JF, Schultz, RA, Ballif, BC, Shaffer, LG. 2012. Haploinsufficiency of SOX5 at 12p12.1 is associated with developmental delays with prominent language delay, behavior problems, and mild dysmorphic features. Hum Mutat, 33: 728-740.

Lasky-Su, J, Neale, BM, Franke, B, Anney, RJ, Zhou, K, Maller, JB, Vasquez, AA, Chen, W, Asherson, P, Buitelaar, J, Banaschewski, T, Ebstein, R, Gill, M, Miranda, A, Mulas, F, Oades, RD, Roeyers, H, Rothenberger, A, Sergeant, J, Sonuga-Barke, E, Steinhausen, HC, Taylor, E, Daly, M, Laird, N, Lange, C, Faraone, SV. 2008. Genome-wide association scan of quantitative traits for attention deficit hyperactivity disorder identifies novel associations and confirms candidate gene associations. Am J Med Genet B Neuropsychiatr Genet, 147B: 1345-1354.

Laumet, G, Chouraki, V, Grenier-Boley, B, Legry, V, Heath, S, Zelenika, D, Fievet, N, Hannequin, D, Delepine, M, Pasquier, F, Hanon, O, Brice, A, Epelbaum, J, Berr, C, Dartigues, J-F, Tzourio, C, Campion, D, Lathrop, M, Bertram, L, Amouyel, P, Lambert, J-C. 2010. Systematic analysis of candidate genes for Alzheimer's disease in a French, genome-wide association study. J Alzheimers Dis, 20: 1181-1188.

Lesch, K-P, Timmesfeld, N, Renner, TJ, Halperin, R, Roser, C, Nguyen, TT, Craig, DW, Romanos, J, Heine, M, Meyer, J, Freitag, C, Warnke, A, Romanos, M, Schafer, H, Walitza, S, Reif, A, Stephan, DA, Jacob, C. 2008. Molecular genetics of adult ADHD: converging evidence from genome-wide association and extended pedigree linkage studies. J Neural Transm, 115: 1573-1585.

Li, MD, Sun, D, Lou, XY, Beuten, J, Payne, TJ, Ma, JZ. 2007. Linkage and association studies in African- and Caucasian-American populations demonstrate that SHC3 is a novel susceptibility locus for nicotine dependence. Mol Psychiatry, 12: 462-473.

Lill, CM, Roehr, JT, McQueen, MB, Kavvoura, FK, Bagade, S, Schjeide, BM, Schjeide, LM, Meissner, E, Zauft, U, Allen, NC, Liu, T, Schilling, M, Anderson, KJ, Beecham, G, Berg, D, Biernacka, JM, Brice, A, DeStefano, AL, Do, CB, Eriksson, N, Factor, SA, Farrer, MJ, Foroud, T, Gasser, T, Hamza, T, Hardy, JA, Heutink, P, Hill-Burns, EM, Klein, C, Latourelle, JC, Maraganore, DM, Martin, ER, Martinez, M, Myers, RH, Nalls, MA, Pankratz, N, Payami, H, Satake, W, Scott, WK, Sharma, M, Singleton, AB, Stefansson, K, Toda, T, Tung, JY, Vance, J, Wood, NW, Zabetian, CP, andMe Genetic Epidemiology of Parkinson's Disease, C, International Parkinson's Disease Genomics, C, Parkinson's Disease, GC, Wellcome Trust Case Control, C, Young, P, Tanzi, RE, Khoury, MJ, Zipp, F, Lehrach, H, Ioannidis, JP, Bertram, L. 2012. Comprehensive research synopsis and systematic meta-analyses in Parkinson's disease genetics: The PDGene database. PLoS Genet, 8: e1002548.

Lv, J, Zhu, P, Yang, Z, Li, M, Zhang, X, Cheng, J, Chen, X, Lu, F. 2015. PCDH20 functions as a tumour-suppressor gene through antagonizing the Wnt/beta-catenin signalling pathway in hepatocellular carcinoma. J Viral Hepat, 22: 199-209.

Ma, W, Tavakoli, T, Derby, E, Serebryakova, Y, Rao, MS, Mattson, MP. 2008. Cell-extracellular matrix interactions regulate neural differentiation of human embryonic stem cells. BMC Dev Biol, 8: 90.

Malinin, NL, Wright, S, Seubert, P, Schenk, D, Griswold-Prenner, I. 2005. Amyloid-beta neurotoxicity is mediated by FISH adapter protein and ADAM12 metalloprotease activity. Proc Natl Acad Sci U S A, 102: 3058-3063.

Mamet, J, Baron, A, Lazdunski, M, Voilley, N. 2002. Proinflammatory mediators, stimulators of sensory neuron excitability via the expression of acid-sensing ion channels. J Neurosci, 22: 10662-10670.

Maretzky, T, Reiss, K, Ludwig, A, Buchholz, J, Scholz, F, Proksch, E, de Strooper, B, Hartmann, D, Saftig, P. 2005. ADAM10 mediates E-cadherin shedding and regulates epithelial cell-cell adhesion, migration, and beta-catenin translocation. Proc Natl Acad Sci U S A, 102: 9182-9187.

Marron, TU, Guerini, V, Rusmini, P, Sau, D, Brevini, TA, Martini, L, Poletti, A. 2005. Androgen-induced neurite outgrowth is mediated by neuritin in motor neurones. J Neurochem, 92: 10-20.

Martin, PJ, Delmotte, MH, Formstecher, P, Lefebvre, P. 2003. PLZF is a negative regulator of retinoic acid receptor transcriptional activity. Nucl Recept, 1: 6.

Martinez-Morales, PL, Quiroga, AC, Barbas, JA, Morales, AV. 2010. SOX5 controls cell cycle progression in neural progenitors by interfering with the WNT-beta-catenin pathway. EMBO Rep, 11: 466-472.

Matthys, A, Van Craenenbroeck, K, Lintermans, B, Haegeman, G, Vanhoenacker, P. 2012. RhoBTB3 interacts with the 5-HT7a receptor and inhibits its proteasomal degradation. Cell Signal, 24: 1053-1063.

Melnick, A, Carlile, GW, McConnell, MJ, Polinger, A, Hiebert, SW, Licht, JD. 2000a. AML-1/ETO fusion protein is a dominant negative inhibitor of transcriptional repression by the promyelocytic leukemia zinc finger protein. Blood, 96: 3939-3947.

Melnick, AM, Westendorf, JJ, Polinger, A, Carlile, GW, Arai, S, Ball, HJ, Lutterbach, B, Hiebert, SW, Licht, JD. 2000b. The ETO protein disrupted in t(8;21)-associated acute myeloid leukemia is a corepressor for the promyelocytic leukemia zinc finger protein. Mol Cell Biol, 20: 2075-2086.

Mick, E, McGough, J, Deutsch, CK, Frazier, JA, Kennedy, D, Goldberg, RJ. 2014. Genome-Wide Association Study of Proneness to Anger. PLoS One, 9: e87257.

Mick, E, Todorov, A, Smalley, S, Hu, X, Loo, S, Todd, RD, Biederman, J, Byrne, D, Dechairo, B, Guiney, A, McCracken, J, McGough, J, Nelson, SF, Reiersen, AM, Wilens, TE, Wozniak, J, Neale, BM, Faraone, SV. 2010. Family-based genome-wide association scan of attention-deficit/hyperactivity disorder. J Am Acad Child Adolesc Psychiatry, 49: 898-905.e893.

Miyamoto-Sato, E, Fujimori, S, Ishizaka, M, Hirai, N, Masuoka, K, Saito, R, Ozawa, Y, Hino, K, Washio, T, Tomita, M, Yamashita, T, Oshikubo, T, Akasaka, H, Sugiyama, J, Matsumoto, Y, Yanagawa, H. 2010. A comprehensive resource of interacting protein regions for refining human transcription factor networks. PLoS One, 5: e9289.

Montoya, ER, Terburg, D, Bos, PA, van Honk, J. 2012. Testosterone, cortisol, and serotonin as key regulators of social aggression: A review and theoretical perspective. Motiv Emot, 36: 65-73.

Morkel, M, Huelsken, J, Wakamiya, M, Ding, J, van de Wetering, M, Clevers, H, Taketo, MM, Behringer, RR, Shen, MM, Birchmeier, W. 2003. Beta-catenin regulates Cripto- and Wnt3-dependent gene expression programs in mouse axis and mesoderm formation. Development, 130: 6283-6294.

Morris, DW, Pearson, RD, Cormican, P, Kenny, EM, O'Dushlaine, CT, Perreault, L-PL, Giannoulatou, E, Tropea, D, Maher, BS, Wormley, B, Kelleher, E, Fahey, C, Molinos, I, Bellini, S, Pirinen, M, Strange, A, Freeman, C, Thiselton, DL, Elves, RL, Regan, R, Ennis, S, Dinan, TG, McDonald, C, Murphy, KC, O'Callaghan, E, Waddington, JL, Walsh, D, O'Donovan, M, Grozeva, D, Craddock, N, Stone, J, Scolnick, E, Purcell, S, Sklar, P, Coe, B, Eichler, EE, Ophoff, R, Buizer, J, Szatkiewicz, J, Hultman, C, Sullivan, P, Gurling, H, McQuillin, A, St Clair, D, Rees, E, Kirov, G, Walters, J, Blackwood, D, Johnstone, M, Donohoe, G, O'Neill, FA, Kendler, KS, Gill, M, Riley, BP, Spencer, CCA, Corvin, A. 2014. An inherited duplication at the gene p21 Protein-Activated Kinase 7 (PAK7) is a risk factor for psychosis. Hum Mol Genet, 23: 3316-3326.

Muller-Tidow, C, Steffen, B, Cauvet, T, Tickenbrock, L, Ji, P, Diederichs, S, Sargin, B, Kohler, G, Stelljes, M, Puccetti, E, Ruthardt, M, deVos, S, Hiebert, SW, Koeffler, HP, Berdel, WE, Serve, H. 2004. Translocation products in acute myeloid leukemia activate the Wnt signaling pathway in hematopoietic cells. Mol Cell Biol, 24: 2890-2904.

Nadri, C, Bersudsky, Y, Belmaker, RH, Agam, G. 2007. Elevated urinary ADAM12 protein levels in lithium-treated bipolar patients. J Neural Transm, 114: 473-477.

Nakamura, T, Muraoka, S, Sanokawa, R, Mori, N. 1998. N-Shc and Sck, two neuronally expressed Shc adapter homologs. Their differential regional expression in the brain and roles in neurotrophin and Src signaling. J Biol Chem, 273: 6960-6967.

Neale, BM, Medland, SE, Ripke, S, Asherson, P, Franke, B, Lesch, KP, Faraone, SV, Nguyen, TT, Schafer, H, Holmans, P, Daly, M, Steinhausen, HC, Freitag, C, Reif, A, Renner, TJ, Romanos, M, Romanos, J, Walitza, S, Warnke, A, Meyer, J, Palmason, H, Buitelaar, J, Vasquez, AA, Lambregts-Rommelse, N, Gill, M, Anney, RJ, Langely, K, O'Donovan, M, Williams, N, Owen, M, Thapar, A, Kent, L, Sergeant, J, Roeyers, H, Mick, E, Biederman, J, Doyle, A, Smalley, S, Loo, S, Hakonarson, H, Elia, J, Todorov, A, Miranda, A, Mulas, F, Ebstein, RP, Rothenberger, A, Banaschewski, T, Oades, RD, Sonuga-Barke, E, McGough, J, Nisenbaum, L, Middleton, F, Hu, X, Nelson, S, Psychiatric, GCAS. 2010. Meta-analysis of genome-wide association studies of attention-deficit/hyperactivity disorder. J Am Acad Child Adolesc Psychiatry, 49: 884-897.

Need, AC, Ge, D, Weale, ME, Maia, J, Feng, S, Heinzen, EL, Shianna, KV, Yoon, W, Kasperaviciute, D, Gennarelli, M, Strittmatter, WJ, Bonvicini, C, Rossi, G, Jayathilake, K, Cola, PA, McEvoy, JP, Keefe, RS, Fisher, EM, St Jean, PL, Giegling, I, Hartmann, AM, Moller, HJ, Ruppert, A, Fraser, G, Crombie, C, Middleton, LT, St Clair, D, Roses, AD, Muglia, P, Francks, C, Rujescu, D, Meltzer, HY, Goldstein, DB. 2009. A genome-wide investigation of SNPs and CNVs in schizophrenia. PLoS Genet, 5: e1000373.

Nikolic, M. 2002. The role of Rho GTPases and associated kinases in regulating neurite outgrowth. Int J Biochem Cell Biol, 34: 731-745.

O'Roak, BJ, Deriziotis, P, Lee, C, Vives, L, Schwartz, JJ, Girirajan, S, Karakoc, E, Mackenzie, AP, Ng, SB, Baker, C, Rieder, MJ, Nickerson, DA, Bernier, R, Fisher, SE, Shendure, J, Eichler, EE. 2011. Exome sequencing in sporadic autism spectrum disorders identifies severe de novo mutations. Nat Genet, 43: 585-589.

O'Roak, BJ, Vives, L, Girirajan, S, Karakoc, E, Krumm, N, Coe, BP, Levy, R, Ko, A, Lee, C, Smith, JD, Turner, EH, Stanaway, IB, Vernot, B, Malig, M, Baker, C, Reilly, B, Akey, JM, Borenstein, E, Rieder, MJ, Nickerson, DA, Bernier, R, Shendure, J, Eichler, EE. 2012. Sporadic autism exomes reveal a highly interconnected protein network of de novo mutations. Nature, 485: 246-250.

O'Shea, PJ, Kim, DW, Logan, JG, Davis, S, Walker, RL, Meltzer, PS, Cheng, SY, Williams, GR. 2012. Advanced bone formation in mice with a dominant-negative mutation in the thyroid hormone receptor beta gene due to activation of Wnt/beta-catenin protein signaling. J Biol Chem, 287: 17812-17822.

Oblander, SA, Brady-Kalnay, SM. 2010. Distinct PTPmu-associated signaling molecules differentially regulate neurite outgrowth on E-, N-, and R-cadherin. Mol Cell Neurosci, 44: 78-93.

Pardon, MC. 2010. Role of neurotrophic factors in behavioral processes: implications for the treatment of psychiatric and neurodegenerative disorders. Vitam Horm, 82: 185-200.

Park, JW, Park, ES, Choi, EN, Park, HY, Jung, SC. 2009. Altered brain gene expression profiles associated with the pathogenesis of phenylketonuria in a mouse model. Clin Chim Acta, 401: 90-99.

Pavlov, KA, Chistiakov, DA, Chekhonin, VP. 2012. Genetic determinants of aggression and impulsivity in humans. J Appl Genet, 53: 61-82.

Pawlowski, JE, Ertel, JR, Allen, MP, Xu, M, Butler, C, Wilson, EM, Wierman, ME. 2002. Liganded androgen receptor interaction with beta-catenin: nuclear co-localization and modulation of transcriptional activity in neuronal cells. J Biol Chem, 277: 20702-20710.

Perche, O, Menuet, A, Marcos, M, Liu, L, Paris, A, Utami, KH, Kervran, D, Cacheux, V, Laudier, B, Briault, S. 2013. Combined deletion of two Condensin II system genes (NCAPG2 and MCPH1) in a case of severe microcephaly and mental deficiency. Eur J Med Genet, 56: 635-641.

Prinzen, C, Trumbach, D, Wurst, W, Endres, K, Postina, R, Fahrenholz, F. 2009. Differential gene expression in ADAM10 and mutant ADAM10 transgenic mice. BMC Genomics, 10: 66.

Puttagunta, R, Schmandke, A, Floriddia, E, Gaub, P, Fomin, N, Ghyselinck, NB, Di Giovanni, S. 2011. RA-RAR-beta counteracts myelin-dependent inhibition of neurite outgrowth via Lingo-1 repression. J Cell Biol, 193: 1147-1156.

Ramklint, M, Stalenheim, EG, von Knorring, A-L, von Knorring, L. 2001. Conduct disorder and personality in a forensic psychiatric population. Eur J Psychiatry, 15: 245-254.

Ramoz, N, Cai, G, Reichert, JG, Silverman, JM, Buxbaum, JD. 2008. An analysis of candidate autism loci on chromosome 2q24-q33: evidence for association to the STK39 gene. Am J Med Genet B Neuropsychiatr Genet, 147B: 1152-1158.

Renou, JP, Bierie, B, Miyoshi, K, Cui, Y, Djiane, J, Reichenstein, M, Shani, M, Hennighausen, L. 2003. Identification of genes differentially expressed in mouse mammary epithelium transformed by an activated beta-catenin. Oncogene, 22: 4594-4610.

Rhee, J, Buchan, T, Zukerberg, L, Lilien, J, Balsamo, J. 2007. Cables links Robo-bound Abl kinase to N-cadherin-bound beta-catenin to mediate Slit-induced modulation of adhesion and transcription. Nat Cell Biol, 9: 883-892.

Rosenfeld, JA, Coppinger, J, Bejjani, BA, Girirajan, S, Eichler, EE, Shaffer, LG, Ballif, BC. 2010. Speech delays and behavioral problems are the predominant features in individuals with developmental delays and 16p11.2 microdeletions and microduplications. J Neurodev Disord, 2: 26-38.

Rubio, ME, Curcio, C, Chauvet, N, Bruses, JL. 2005. Assembly of the N-cadherin complex during synapse formation involves uncoupling of p120-catenin and association with presenilin 1. Mol Cell Neurosci, 30: 118-130.

Sagazio, A, Shohreh, R, Salvatori, R. 2011. Effects of GH deficiency and GH replacement on inter-male aggressiveness in mice. Growth Horm IGF Res, 21: 76-80.

Sanchez-Mora, C, Ramos-Quiroga, JA, Bosch, R, Corrales, M, Garcia-Martinez, I, Nogueira, M, Pagerols, M, Palomar, G, Richarte, V, Vidal, R, Arias-Vasquez, A, Bustamante, M, Forns, J, Gross-Lesch, S, Guxens, M, Hinney, A, Hoogman, M, Jacob, C, Jacobsen, KK, Kan, CC, Kiemeney, L, Kittel-Schneider, S, Klein, M, Onnink, M, Rivero, O, Zayats, T, Buitelaar, J, Faraone, SV, Franke, B, Haavik, J, Johansson, S, Lesch, KP, Reif, A, Sunyer, J, Bayes, M, Casas, M, Cormand, B, Ribases, M. 2015. Case-control genome-wide association study of persistent attention-deficit hyperactivity disorder identifies FBXO33 as a novel susceptibility gene for the disorder. Neuropsychopharmacology, 40: 915-926.

Schanze, I, Schanze, D, Bacino, CA, Douzgou, S, Kerr, B, Zenker, M. 2013. Haploinsufficiency of SOX5, a member of the SOX (SRY-related HMG-box) family of transcription factors is a cause of intellectual disability. Eur J Med Genet, 56: 108-113.

Seetharaman, A, Selman, G, Puckrin, R, Barbier, L, Wong, E, D'Souza, SA, Roy, PJ. 2011. MADD-4 is a secreted cue required for midline-oriented guidance in Caenorhabditis elegans. Dev Cell, 21: 669-680.

Sengle, G, Tsutsui, K, Keene, DR, Tufa, SF, Carlson, EJ, Charbonneau, NL, Ono, RN, Sasaki, T, Wirtz, MK, Samples, JR, Fessler, LI, Fessler, JH, Sekiguchi, K, Hayflick, SJ, Sakai, LY. 2012. Microenvironmental regulation by fibrillin-1. PLoS Genet, 8: e1002425.

Shan, WS, Tanaka, H, Phillips, GR, Arndt, K, Yoshida, M, Colman, DR, Shapiro, L. 2000. Functional cis-heterodimers of N- and R-cadherins. J Cell Biol, 148: 579-590.

Shea, TB, Beermann, ML, Leli, U, Nixon, RA. 1992. Opposing influences of protein kinase activities on neurite outgrowth in human neuroblastoma cells: initiation by kinase A and restriction by kinase C. J Neurosci Res, 33: 398-407.

So, PL, Yip, PK, Bunting, S, Wong, LF, Mazarakis, ND, Hall, S, McMahon, S, Maden, M, Corcoran, JP. 2006. Interactions between retinoic acid, nerve growth factor and sonic hedgehog signalling pathways in neurite outgrowth. Dev Biol, 298: 167-175.

Stalenheim, EG. 2004. Long-term validity of biological markers of psychopathy and criminal recidivism: follow-up 6-8 years after forensic psychiatric investigation. Psychiatry Res, 121: 281-291.

Stefansson, H, Ophoff, RA, Steinberg, S, Andreassen, OA, Cichon, S, Rujescu, D, Werge, T, Pietilainen, OP, Mors, O, Mortensen, PB, Sigurdsson, E, Gustafsson, O, Nyegaard, M, Tuulio-Henriksson, A, Ingason, A, Hansen, T, Suvisaari, J, Lonnqvist, J, Paunio, T, Borglum, AD, Hartmann, A, Fink-Jensen, A, Nordentoft, M, Hougaard, D, Norgaard-Pedersen, B, Bottcher, Y, Olesen, J, Breuer, R, Moller, HJ, Giegling, I, Rasmussen, HB, Timm, S, Mattheisen, M, Bitter, I, Rethelyi, JM, Magnusdottir, BB, Sigmundsson, T, Olason, P, Masson, G, Gulcher, JR, Haraldsson, M, Fossdal, R, Thorgeirsson, TE, Thorsteinsdottir, U, Ruggeri, M, Tosato, S, Franke, B, Strengman, E, Kiemeney, LA, Genetic, R, Outcome in, P, Melle, I, Djurovic, S, Abramova, L, Kaleda, V, Sanjuan, J, de Frutos, R, Bramon, E, Vassos, E, Fraser, G, Ettinger, U, Picchioni, M, Walker, N, Toulopoulou, T, Need, AC, Ge, D, Yoon, JL, Shianna, KV, Freimer, NB, Cantor, RM, Murray, R, Kong, A, Golimbet, V, Carracedo, A, Arango, C, Costas, J, Jonsson, EG, Terenius, L, Agartz, I, Petursson, H, Nothen, MM, Rietschel, M, Matthews, PM, Muglia, P, Peltonen, L, St Clair, D, Goldstein, DB, Stefansson, K, Collier, DA. 2009. Common variants conferring risk of schizophrenia. Nature, 460: 744-747.

Stergiakouli, E, Hamshere, M, Holmans, P, Langley, K, Zaharieva, I, Hawi, Z, Kent, L, Gill, M, Williams, N, Owen, MJ, O'Donovan, M, Thapar, A. 2012. Investigating the contribution of common genetic variants to the risk and pathogenesis of ADHD. Am J Psychiatry, 169: 186-194.

Suzuki, N, Numakawa, T, Chou, J, de Vega, S, Mizuniwa, C, Sekimoto, K, Adachi, N, Kunugi, H, Arikawa-Hirasawa, E, Yamada, Y, Akazawa, C. 2014. Teneurin-4 promotes cellular protrusion formation and neurite outgrowth through focal adhesion kinase signaling. FASEB J, 28: 1386-1397.

Szklarczyk, A, Conant, K, Owens, DF, Ravin, R, McKay, RD, Gerfen, C. 2007. Matrix metalloproteinase-7 modulates synaptic vesicle recycling and induces atrophy of neuronal synapses. Neuroscience, 149: 87-98.

Tan, MS, Yu, JT, Jiang, T, Zhu, XC, Wang, HF, Zhang, W, Wang, YL, Jiang, W, Tan, L. 2013. NLRP3 polymorphisms are associated with late-onset Alzheimer's disease in Han Chinese. J Neuroimmunol, 265: 91-95.

Tielbeek, JJ, Medland, SE, Benyamin, B, Byrne, EM, Heath, AC, Madden, PA, Martin, NG, Wray, NR, Verweij, KJ. 2012. Unraveling the genetic etiology of adult antisocial behavior: a genome-wide association study. PLoS One, 7: e45086.

Tobaben, S, Sudhof, TC, Stahl, B. 2000. The G protein-coupled receptor CL1 interacts directly with proteins of the Shank family. J Biol Chem, 275: 36204-36210.

Trent, S, Drew, CJ, Mitchell, PJ, Bailey, SJ. 2009. Chronic treatment with 13-cis-retinoic acid changes aggressive behaviours in the resident-intruder paradigm in rats. Eur Neuropsychopharmacol, 19: 876-886.

UniProt, C. 2014. Activities at the Universal Protein Resource (UniProt). Nucleic Acids Res, 42: D191-198.

Votin, V, Nelson, WJ, Barth, AI. 2005. Neurite outgrowth involves adenomatous polyposis coli protein and beta-catenin. J Cell Sci, 118: 5699-5708.

Walter, IB. 1996. Triiodothyronine exerts a trophic action on rat sensory neuron survival and neurite outgrowth through different pathways. Eur J Neurosci, 8: 455-466.

Wang, JY, Darbinyan, A, White, MK, Darbinian, N, Reiss, K, Amini, S. 2014. Involvement of IRS-1 interaction with ADAM10 in the regulation of neurite extension. J Cell Physiol, 229: 1039-1046.

Wang, X, Chow, FL, Oka, T, Hao, L, Lopez-Campistrous, A, Kelly, S, Cooper, S, Odenbach, J, Finegan, BA, Schulz, R, Kassiri, Z, Lopaschuk, GD, Fernandez-Patron, C. 2009. Matrix metalloproteinase-7 and ADAM-12 (a disintegrin and metalloproteinase-12) define a signaling axis in agonist-induced hypertension and cardiac hypertrophy. Circulation, 119: 2480-2489.

Weick, JP, Austin Johnson, M, Zhang, SC. 2009. Developmental regulation of human embryonic stem cell-derived neurons by calcium entry via transient receptor potential channels. Stem Cells, 27: 2906-2916.

Wong, LE, Reynolds, AB, Dissanayaka, NT, Minden, A. 2010. p120-catenin is a binding partner and substrate for Group B Pak kinases. J Cell Biochem, 110: 1244-1254.

Woods, CG, Bond, J, Enard, W. 2005. Autosomal recessive primary microcephaly (MCPH): a review of clinical, molecular, and evolutionary findings. Am J Hum Genet, 76: 717-728.

Wright, KM, Lyon, KA, Leung, H, Leahy, DJ, Ma, L, Ginty, DD. 2012. Dystroglycan organizes axon guidance cue localization and axonal pathfinding. Neuron, 76: 931-944.

Wu, D, Huang, W, Richardson, PM, Priestley, JV, Liu, M. 2008. TRPC4 in rat dorsal root ganglion neurons is increased after nerve injury and is necessary for neurite outgrowth. J Biol Chem, 283: 416-426.

Wu, Q, Dawson, MI, Zheng, Y, Hobbs, PD, Agadir, A, Jong, L, Li, Y, Liu, R, Lin, B, Zhang, XK. 1997. Inhibition of trans-retinoic acid-resistant human breast cancer cell growth by retinoid X receptor-selective retinoids. Mol Cell Biol, 17: 6598-6608.

Wu, X, Hepner, K, Castelino-Prabhu, S, Do, D, Kaye, MB, Yuan, XJ, Wood, J, Ross, C, Sawyers, CL, Whang, YE. 2000. Evidence for regulation of the PTEN tumor suppressor by a membrane-localized multi-PDZ domain containing scaffold protein MAGI-2. Proc Natl Acad Sci U S A, 97: 4233-4238.

Xu, J, Paquet, M, Lau, AG, Wood, JD, Ross, CA, Hall, RA. 2001. beta 1-adrenergic receptor association with the synaptic scaffolding protein membrane-associated guanylate kinase inverted-2 (MAGI-2). Differential regulation of receptor internalization by MAGI-2 and PSD-95. J Biol Chem, 276: 41310-41317.

Xu, X, Harder, J, Flynn, DC, Lanier, LM. 2009. AFAP120 regulates actin organization during neuronal differentiation. Differentiation, 77: 38-47.

Yanagisawa, H, Komuta, Y, Kawano, H, Toyoda, M, Sango, K. 2010. Pleiotrophin induces neurite outgrowth and up-regulates growth-associated protein (GAP)-43 mRNA through the ALK/GSK3beta/beta-catenin signaling in developing mouse neurons. Neurosci Res, 66: 111-116.

Yang, L, Neale, BM, Liu, L, Lee, SH, Wray, NR, Ji, N, Li, H, Qian, Q, Wang, D, Li, J, Faraone, SV, Wang, Y, Doyle, AE, Reif, A, Rothenberger, A, Franke, B, Sonuga-Barke, EJ, Steinhausen, HC, Buitelaar, JK, Kuntsi, J, Biederman, J, Lesch, KP, Kent, L, Asherson, P, Oades, RD, Loo, SK, Nelson, SF, Faraone, SV, Smalley, SL, Banaschewski, T, Arias Vasquez, A, Todorov, A, Charach, A, Miranda, A, Warnke, A, Thapar, A, Neale, BM, Cormand, B, Freitag, C, Mick, E, Mulas, F, Middleton, F, HakonarsonHakonarson, H, Palmason, H, Schafer, H, Roeyers, H, McGough, JJ, Romanos, J, Crosbie, J, Meyer, J, Ramos-Quiroga, JA, Sergeant, J, Elia, J, Langely, K, Nisenbaum, L, Romanos, M, Daly, MJ, Ribases, M, Gill, M, O'Donovan, M, Owen, M, Casas, M, Bayes, M, Lambregts-Rommelse, N, Williams, N, Holmans, P, Anney, RJ, Ebstein, RP, Schachar, R, Medland, SE, Ripke, S, Walitza, S, Nguyen, TT, Renner, TJ, Hu, X. 2013. Polygenic transmission and complex neuro developmental network for attention deficit hyperactivity disorder: genome-wide association study of both common and rare variants. Am J Med Genet B Neuropsychiatr Genet, 162b: 419-430.

Zamir, I, Dawson, J, Lavinsky, RM, Glass, CK, Rosenfeld, MG, Lazar, MA. 1997. Cloning and characterization of a corepressor and potential component of the nuclear hormone receptor repression complex. Proc Natl Acad Sci U S A, 94: 14400-14405.

Zayats, T, Athanasiu, L, Sonderby, I, Djurovic, S, Westlye, LT, Tamnes, CK, Fladby, T, Aase, H, Zeiner, P, Reichborn-Kjennerud, T, Knappskog, PM, Knudsen, GP, Andreassen, OA, Johansson, S, Haavik, J. 2015. Genome-wide analysis of attention deficit hyperactivity disorder in norway. PLoS One, 10: e0122501.
